# The FACT-targeted drug CBL0137 enhances the effects of rituximab to inhibit B-cell non-Hodgkin’s lymphoma tumor growth by promoting apoptosis and autophagy

**DOI:** 10.1186/s12964-022-01031-x

**Published:** 2023-01-23

**Authors:** Yan Lv, Yuxin Du, Kening Li, Xiao Ma, Juan Wang, Tongde Du, Yuxin Ma, Yue Teng, Weiyan Tang, Rong Ma, Jianqiu Wu, Jianzhong Wu, Jifeng Feng

**Affiliations:** 1 Nanjing Medical University Affiliated Cancer Hospital, Jiangsu Cancer Hospital, Jiangsu Institute of Cancer Research, 42 Baiziting, Nanjing, 210009 Jiangsu Province China; 2 Center for Global Health, School of Public Health, Nanjing Medical University, Nanjing, 211166 Jiangsu Province China; 3 Department of General Surgery, The Affiliated Zhongda Hospital of Southeast University, 87 Dingjiaqiao, Nanjing, 210009 Jiangsu Province China

**Keywords:** CBL0137, Rituximab, B-cell non-Hodgkin’s lymphoma, FACT, Apoptosis, Autophagy

## Abstract

**Background:**

Aggressive B-cell non-Hodgkin’s lymphoma (B-NHL) patients often develop drug resistance and tumor recurrence after conventional immunochemotherapy, for which new treatments are needed.

**Methods:**

We investigated the antitumor effects of CBL0137. In vitro, cell proliferation was assessed by CCK-8 and colony formation assay. Flow cytometry was performed to analyze cell cycle progression, apoptosis, mitochondrial depolarization, and reactive oxygen species (ROS) production. Autophagy was detected by transmission electron microscopy and mGFP-RFP-LC3 assay, while western blotting was employed to detect proteins involved in apoptosis and autophagy. RNA-sequencing was conducted to analyze the transcription perturbation after CBL0137 treatment in B-NHL cell lines. Finally, the efficacy and safety of CBL0137, rituximab, and their combination were tested in vivo.

**Results:**

CBL0137, a small molecule anticancer agent that has significant antitumor effects in B-NHL. CBL0137 sequesters the FACT (facilitates chromatin transcription) complex from chromatin to produce cytotoxic effects in B-NHL cells. In addition, we discovered novel anticancer mechanisms of CBL0137. CBL0137 inhibited human B-NHL cell proliferation by inducing cell cycle arrest in S phase via the c-MYC/p53/p21 pathway. Furthermore, CBL0137 triggers ROS generation and induces apoptosis and autophagy in B-NHL cells through the ROS-mediated PI3K/Akt/mTOR and MAPK signaling pathways. Notably, a combination of CBL0137 and rituximab significantly suppressed B-NHL tumor growth in subcutaneous models, consistent with results at the cellular level in vitro.

**Conclusions:**

CBL0137 has potential as a novel approach for aggressive B-NHL, and its combination with rituximab can provide new therapeutic options for patients with aggressive B-NHL.

**Video Abstract**

**Supplementary Information:**

The online version contains supplementary material available at 10.1186/s12964-022-01031-x.

## Background

Non-Hodgkin’s lymphoma (NHL) is a heterogeneous disease caused by the malignant transformation of lymphocytes, 60–80% of which originate from B cells. Aggressive B-cell NHL (B-NHL) is the most common subtype of NHL and mainly includes diffuse large B-cell lymphoma (DLBCL), Burkitt lymphoma (BL), and mantle cell lymphoma (MCL) [1, 2]. Current standard treatment options for B-NHL include anti-CD20 antibody rituximab (R-) plus CHOP (cyclophosphamide, doxorubicin, vincristine, and prednisone) chemotherapy, hematopoietic stem cell transplantation, and chimeric antigen receptor (CAR) T-cell therapy. However, despite this intensive multimodal regime, 30% to 50% of B-NHL patients are still resistant to treatment, leading to disease recurrence and progression [3, 4]. Moreover, CAR-T therapies are associated with an increased risk of infection and acute and chronic severe physical impairment [5]. Therefore, the development of more efficacious and safer new drugs for relapsed/refractory B-NHL patients is still a highly unmet need.

CBL0137 is a novel nongenotoxic anticancer drug belonging to a family of small molecules called curaxins [6], which exhibits antitumor activity by targeting FACT (facilitates chromatin transcription), a histone chaperone that is highly expressed and associated with increased aggressiveness, stemness, and poor prognosis in a variety of cancer types [6–18]. FACT is composed of 2 subunits, structure-specific recognition protein 1 (SSRP1) and suppressor of Ty-16 (SPT16), which promotes transcriptional elongation by facilitating nucleosome disassembly and reassembly and is involved in the regulation of DNA replication and DNA damage repair [19, 20]. Chromatin trapping induced by CBL0137 exhausts cellular FACT, thereby mediating a series of downstream effects, including p53 activation and NF-κB inhibition [6, 11]. In addition, CBL0137 also inhibits the self-renewal of cancer stem cells or tumor-initiating cells via NOTCH1 activation [16]. Currently, CBL0137 is undergoing several clinical trials for hematological cancers and solid tumors (ClinicalTrials.gov Identifier: NCT01905228, NCT02931110, and NCT03727789). Therefore, CBL0137 can be considered a candidate for monotherapy while also exerting advantages in combination therapy, which provides the effectiveness of chemotherapy and the benefits of targeted therapy, giving it more significant potential and clinical significance.

Rituximab is a chimeric monoclonal antibody targeted against the pan-B-cell marker CD20. Immunotherapy represented by rituximab has a positive efficacy in treating B-NHL patients, but its primary or acquired resistance seriously affects the long-term survival benefits [21, 22]. In modern medicine, it is urgent to explore the “one plus one is greater than two” combination therapy of novel biologics and targeted therapies, which can enhance efficacy over what has been shown with monotherapy and further improve the prognosis.

In this study, firstly, we have shown that CBL0137 has a significant inhibitory effect on B-NHL tumor growth mainly by inducing apoptosis and autophagy. Additionally, we also demonstrated that CBL0137 combined with rituximab enhances tumor growth inhibition in vitro and in vivo, and this combination regimen may have clinical benefits for B-NHL patients.

## Materials and methods

### Cell lines and cell culture

The B-NHL cell lines SU-DHL-4, Farage, Raji, and Jeko-1 were purchased from American Type Culture Collection (ATCC). All cell lines were cultured in RPMI-1640 medium (Thermo Fisher Scientific, USA) supplemented with 10% heat-inactivated fetal bovine serum (Gibco, USA) at 37 °C in a 5% CO_2_ humidified incubator. All cell lines were authenticated and routinely tested for mycoplasma contamination.

### Reagents and antibodies

Reagents and antibodies are detailed in the Supplemental Methods (Additional file 1: Table S1 and S2).

### Cell proliferation assay figures

Cell proliferation was measured by cell counting kit-8 (CCK-8) assays (APE × BIO, USA) according to the manufacturer’s instructions. The calculation methods for the IC_50_ and synergy scores based on the Bliss assay are detailed in the Additional file 1: Methods.

### Cytotoxicity assay

The cytotoxicity under different treatments was assessed by using a lactate dehydrogenase (LDH) release kit (Beyotime, China). The studies were carried out in accordance with the instructions provided by the supplier.

### Colony formation assay

B-NHL cells were mixed with an equal volume of methylcellulose colony assay medium (Stem Cell Technologies, Canada), plated in 12-well plates, and incubated for two weeks. Colonies with a diameter of more than 0.1 mm were calculated.

### Cell cycle and apoptosis analysis

For cell cycle analysis, B-NHL cells were treated with CBL0137 (0.5 μM, 1.0 μM, 1.5 μM, and 2.0 μM) for 24 h and stained with PI/RNase (KeyGEN BioTECH, China) for 30 min. An Annexin V-FITC/PI Apoptosis Kit from Vazyme was used for apoptosis analysis. Flow cytometry was used for detection, and the data were analyzed by FlowJo software.

### Analysis of reactive oxygen species (ROS) and mitochondrial membrane (Δψm) levels by flow cytometry in B-NHL cells

ROS were determined by the Reactive Oxygen Species Assay Kit (Solarbio, China). The Δψm was determined using a JC-1 Staining Dye Assay Kit (Solarbio, China) according to the manufacturer’s instructions. Briefly, the cells were observed by fluorescence microscopy and analyzed by flow cytometry. The experimental details are provided in the Supplemental Methods.

### DNA fragmentation analysis

The formation of DNA fragments during apoptosis can be detected with a DNA Ladder Extraction Kit with Spin Column (Beyotime, China). DNA fragments were finally detected by 1.5% agarose gel electrophoresis.

### Western blotting

B-NHL cells were lysed in RIPA lysis buffer (Beyotime, China) after CBL0137 treatment. Cytoplasmic and nuclear proteins were prepared using a Nuclear and Cytoplasmic Protein Extraction Kit (Beyotime, China). Equal amounts of protein extracts were separated by SDS-PAGE (GenScript ProBio, USA) and transferred to nitrocellulose membranes (Cytiva, Germany). The protein bands were visualized by enhanced chemiluminescence (Millipore, USA) and analyzed using ImageJ software. The experimental details are provided in the Supplemental Methods.

### Transmission electron microscopy

SU-DHL-4, Raji, and Jeko-1 cells were treated with CBL0137 (2.0 μM) for 24 h. Cells were collected and then fixed, dehydrated, embedded, sectioned, and stained as previously described [23]. Finally, the cell morphology was observed under a JEM-1200 transmission electron microscopy (JEOL, Japan).

### Autophagic flux analysis by immunofluorescence staining (RFP-GFP-LC3 assay)

To observe autophagosomes and autolysosomes and to analyze autophagic flux, mRFP-GFP-LC3B adenovirus (HANBIO, China) was used for transduction according to the instructions. Twenty-four hours after transduction, the cells were cultured for 24 h with or without CBL0137 (1.0 μM or 2.0 μM) and then scanned by fluorescence microscopy. The red fluorescence spots (mRFP alone) indicated autolysosomes, and yellow fluorescence spots (overlay of mRFP and GFP) indicated early autophagosomes.

### RNA extraction, real-time quantitative polymerase chain reaction (PCR), and RNA sequencing

The primers used for these experimental assays are listed in Additional file 1: Table S3, and details are available in the Supplemental Methods.

### Mouse studies

All animal experiments were approved by the Institutional Animal Care and Use Committee of Nanjing Medical University (approval no. IACUC-2106022) and were conducted following the guidelines of this committee. The subcutaneous injection model and evaluation of the anticancer effects of CBL0137 are described in the Supplemental Methods.

### Statistical analysis

We performed all experiments in triplicate. GraphPad Prism 8.0 and SPSS 25.0 software were used for statistical analyses. All data are expressed as the mean ± standard deviation (SD). *P < 0.05, **P < 0.01, ***P < 0.001, and ****P < 0.0001 were considered statistically significant.

## Results

### FACT is a therapeutic target in B-NHL

**Fig. 1:**
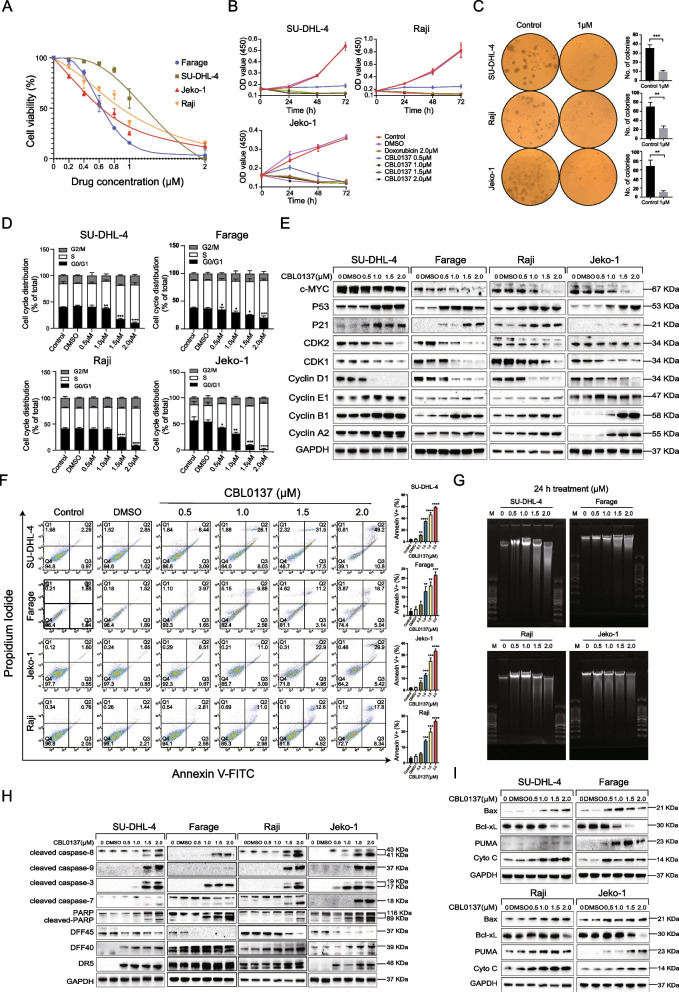
CBL0137 inhibits B-NHL cell proliferation by inducing cell cycle arrest and promoting apoptosis. **A** Cell viability of B-NHL cells treated with various concentrations of CBL0137 for 24 h was measured by CCK-8 assay. **B** B-NHL cells were treated with 0.5 μM, 1.0 μM, 1.5 μM, 2.0 μM CBL0137, and 2.0 μM doxorubicin for 24 h, 48 h, and 72 h. The absorbance values at 450 nm were determined. **C** Cell proliferation was detected by colony formation assay after 24 h treatment with CBL0137 (1.0 μM). **D** The cell cycle was determined by flow cytometry. The percentages of cell cycle phases were shown in the bar chart. CBL0137 induced a significant increase in the proportion of B-NHL cells in S phase. **E** The expression of cyclins, CDKs, c-MYC, p21, and p53 was detected by western blotting in B-NHL cells treated with CBL0137. **F** Four B-NHL cells (SU-DHL-4, Farage, Raji, and Jeko-1) were treated with specified concentrations of CBL0137 for 24 h. Cell apoptosis was assessed by the Annexin V-FITC Apoptosis Detection Kit and flow cytometry. Representative results are shown on the left and statistical results are shown on the right. **G** B-NHL cells were treated with different concentrations of CBL0137 for 24 h and then were collected, and chromosomal DNA was isolated and purified using DNA Ladder Detection Kit according to manufacturer's instructions to explore the effect of CBL0137 on DNA fragmentation in B-NHL cells. **H**, **I** Representative proteins expression in four B-NHL cells treated with different concentrations of CBL0137 and vehicle control (DMSO) for 24 h to evaluate the status of apoptotic cascade and mitochondrial apoptosis. The data are representative and taken from one of three independent experiments. The data results are expressed as mean ± SD (*P < 0.05, **P < 0.01, ***P < 0.001, ****P < 0.0001, Student’s t-test)

To evaluate the potential role of FACT in B-NHL, we first examined the transcript level of FACT complex subunits in the TCGA database. As shown in Additional file 1: Fig. S1A, SSRP1 and SUPT16H mRNA levels were significantly upregulated in DLBCL compared with normal tissue (NT), and SSRP1 and SPT16 were significantly correlated (R = 0.73, *P* = 4.2e-09) (Additional file 1: Fig. S1B). At the protein level, the expression of SSRP and SUPT16H were both increased in B-NHL cell lines compared with the healthy donor (Additional file 1: Fig. S1C). To understand the functional significance of high FACT expression, we compared the sensitivity of different B-NHL cell lines to FACT inhibition with CBL0137 treatment. Cell proliferation assays showed that CBL0137 significantly inhibited B-NHL cell proliferation in a concentration and time-dependent manner. The IC_50_ values of CBL0137 among B-NHL cell lines were different due to the heterogeneity among cell lines. (Fig. 1A, B and Additional file 1: Fig. S1D–F). The colony formation ability of B-NHL cells was also inhibited after CBL0137 treatment (Fig. 1C, Additional file 1: Fig. S1G). These results indicate that CBL0137 can effectively inhibit B-NHL cell proliferation in a certain range of drug concentrations.

Western blotting showed that increasing concentrations of CBL0137 induced relocalization of the FACT subunits SSRP1 and SUPT16H from the cytosol (soluble fraction) to the chromatin-containing fraction (pellet) of treated cells (Additional file 1: Fig. S1H). p53 and phosphor (p)-p53 were significantly activated in a CBL0137-dose-dependent manner, while NF-κB-mediated transcriptional activity was inhibited (Additional file 1: Fig. S1I). Thus, the established anticancer mechanism of CBL0137 also applies to B-NHL.

### CBL0137 induces B-NHL cell cycle arrest in S phase by regulating the c-MYC/p53/21 pathway

Cell proliferation inhibition is usually accompanied by changes in cell cycle progression. RNA-seq data showed that the cell cycle signaling pathway was significantly altered after CBL0137 treatment in B-NHL cells (Additional file 1: Fig. S2A). Moreover, the flow cytometry results showed that with increasing concentration, the cell population in G0/G1 phase was decreased, accompanied by an increase in the population in S phase in B-NHL cells, while there was no significant change in G2 phase (Fig. 1D, Additional file 1: Fig. S2B). The cell cycle is precisely regulated by a variety of proteins, including cyclins and cyclin-dependent kinases (CDKs) [24]. We further assessed the expression of proteins involved in the cell cycle by western blotting (Fig. 1E); cyclin D1, CDK1, and CDK2 expression was significantly decreased, and cyclin E1, cyclin B2, and cyclin A2 expression was upregulated to varying degrees after treatment with CBL0137 for 24 h in B-NHL cells. These results suggested that CBL0137 not only induces significant S phase arrest but also regulates the expression of G1/S phase-associated proteins.

c-MYC has been shown to play a critical role in the cell cycle by regulating the activation of p53 and p21 [25, 26]. Our results further confirmed that CBL0137 could inhibit c-MYC expression and promote the upregulation of p53 and p21, thus regulating the expression of cell cycle-related proteins (Fig. 1E).

### CBL0137 promotes cell apoptosis in B-NHL cells by activating extrinsic and intrinsic apoptotic pathways

We next investigated whether there were other potential mechanisms by which CBL0137 inhibits B-NHL cell growth. Flow cytometry analysis showed that CBL0137 induced a significant increase in both early (located in quadrant Q3) and late apoptotic (located in quadrant Q2) cell subsets in a concentration-dependent manner for B-NHL cell lines. In particular, 2.0 μM CBL0137 induced apoptosis in approximately 60% of SU-DHL-4 cells (Fig. 1F). The terminal stage of apoptosis is characterized by nucleosomal DNA fragmentation [27], and our results were also observed in the 1.5 μM and 2.0 μM groups (Fig. 1G).

Studies have shown that apoptosis can be mediated either by extrinsic or intrinsic pathways [28, 29]. We further investigated the molecular pathways involved in CBL0137-induced apoptosis by western blotting. Our results showed that the expression levels of cleaved caspase-8 and its downstream targets, caspase-3, caspase-7, and PARP, as well as DR5, were elevated (Fig. 1H). Moreover, the opposite expression patterns of DFF45 and DFF40 promoted DNA fragmentation (a hallmark of apoptosis), suggesting that CBL0137 induces extrinsic apoptotic pathways. In addition, we also detected changes in the expression of the Bcl-2 family and mitochondrial damage-related proteins (Fig. 1I). The results showed that cleaved caspase-9, the proapoptotic proteins Bax and PUMA, and cytochrome C were increased, whereas the antiapoptotic protein Bcl-xL was decreased. Similar results were also found in a time-dependent manner (Additional file 1: Fig. S2C). All the above results suggest that CBL0137 activates both caspase-dependent extrinsic and intrinsic apoptotic pathways in B-NHL cells.

### CBL0137 induces autophagy in B-NHL cells

**Fig. 2:**
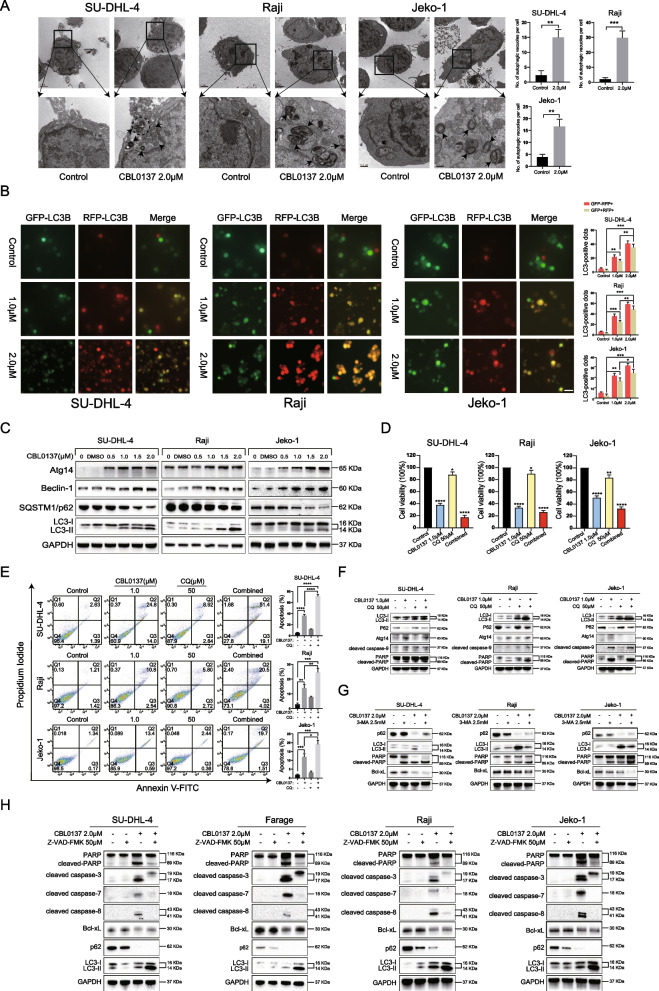
CBL0137 induces protective autophagy in B-NHL cells. **A** Autophagosomes in SU-DHL-4, Raji, and Jeko-1 cells treated with or without 2.0 μM CBL0137 were observed by TEM. The arrow indicates the autophagosome. Scale bars = 2 μm. **B** The expression of GFP + /mRFP + (yellow) and GFP-/mRFP + (red) LC3 puncta was observed by fluorescence microscopy in B-NHL cells treated with or without 1.0 μM or 2.0 μM CBL0137 for 24 h. Representative images and quantitative analysis of fluorescent LC3 puncta were shown. Scale bars = 30 μm. **C** Expression of autophagy-related proteins was detected by western blotting in B-NHL cells treated with CBL0137 or DMSO. **D**–**F** Effect of chloroquine (CQ) on CBL0137-mediated autophagy, cell viability, and apoptosis. Cells were pretreated with 50 μM CQ for 1 h and then exposed to 1.0 μM CBL0137. **D** Cell viability was determined by CCK-8. **E** Apoptosis was measured by flow cytometry. **F** Conversions of LC3I to LC3II, p62, Atg14, and PARP were examined by western blotting. **G** B-NHL cells were pretreated with 2.5 mM 3-methyladenine (3-MA) for 1 h and then exposed to 2.0 μM CBL0137. The expression of PARP, p62, LC3, Bcl-xL was detected by western blotting. **H** 50 μM Z-VAD-FMK was pretreated for 1 h and then exposed to 2.0 μM CBL0137. Caspase-related proteins and autophagy-related proteins were detected by using western blotting

According to previous studies, autophagy has been reported to be closely related to apoptosis, thereby determining cell survival and death [30, 31]. Our RNA-seq data showed that autophagy-related pathways in Jeko-1 cells were significantly upregulated after CBL0137 treatment (Additional file 1: Fig. S3A). Therefore, we first analyzed the ultrastructure of B-NHL cell lines treated with 2.0 μM CBL0137 by transmission electron microscopy (TEM) and showed that more double-membrane structures could be observed in the cytoplasm in the treated group than in the control group (Fig. 2A). Autophagy is a dynamic process. To explore whether CBL0137 could increase autophagic flux in B-NHL cell lines, we transduced B-NHL cells with mRFP-GFP-LC3B adenovirus particles and monitored autophagy. According to the operating instructions and technical principles, the LC3-positive autolysosomes were marked with red puncta, and autophagosomes were marked with yellow puncta [32]. After incubating with CBL0137 for 24 h, images were obtained by fluorescence microscopy. As shown in Fig. 2B, GFP-RFP-LC3 positive autophagosomes were significantly increased in B-NHL cells with CBL0137 treatment in the merged pictures and increased with increasing concentration, suggesting that CBL0137 induced autophagosome-lysosome fusion in B-NHL cells. Western blotting further verified the levels of LC3, SQSTM1/p62, Atg14, and Beclin-1 in B-NHL cells treated with CBL0137 (Fig. 2C, Additional file 1: Fig. S3B, C). Taken together, these findings demonstrated that CBL0137 is an effective autophagy inducer in human B-NHL cells.

### Inhibition of autophagy promoted CBL0137-induced apoptosis in B-NHL cells

Activation of apoptosis is a common event in tumor cells in response to autophagy induction [33, 34]. To investigate whether there was a causal relationship between CBL0137-induced autophagy and apoptosis, we used chloroquine (CQ) and 3-methyladenine (3-MA) to block autophagy in CBL0137-treated B-NHL cells. Our data showed that in the presence of CQ, cell viability after CBL0137 treatment was significantly reduced (Fig. 2D, Additional file 1: Fig. S3D). The flow cytometry results indicated that the apoptosis rates of B-NHL cells were significantly higher in the CQ combined with CBL0137 B-NHL cells than in the CBL0137 single treatment group (Fig. 2E). We used CCK-8 and flow cytometry assays, and both results suggested that CBL0137 in combination with autophagy inhibitors could further promote cell death. Further western blotting confirmed the enhanced CBL0137-induced accumulation of LC3II and decreased degradation of SQSTM1/p62 and Atg14 after CQ blockade. Notably, CQ-pretreated SU-DHL-4 and Raji cells increased CBL0137-mediated cleavage of PARP and caspase-9 (Fig. 2F). Moreover, 3-MA pretreatment not only inhibited autophagy in B-NHL cells but also further promoted apoptosis (Fig. 2G). Collectively, these findings suggest that the inhibition of autophagy enhances CBL0137-induced apoptosis in B-NHL cells.

To further verify the relationship between apoptosis and autophagy. We used an irreversible pan-caspase inhibitor, Z-VAD-FMK (Fig. 2H). Z-VAD-FMK in combination with CBL0137 significantly decreased the cleaved forms of PARP, caspase-3, caspase-7, and caspase-8 in four B-NHL cell lines and reversed the reduced expression of Bcl-xL induced by CBL0137 alone in Farage cells. These results indicated that CBL0137-induced apoptosis was caspase-dependent. Furthermore, we found that the combination of Z-VAD-FMK and CBL0137 significantly increased the accumulation of LC3II, and the consumption of p62 was further increased, suggesting that inhibition of apoptosis promoted autophagy. CBL0137-induced autophagy is a cell survival mechanism; blocking autophagy increases cell death, while inhibiting apoptosis promotes the survival of B-NHL cells.

### CBL0137-induced apoptosis and autophagy are dependent on mitochondrial ROS generation

**Fig. 3:**
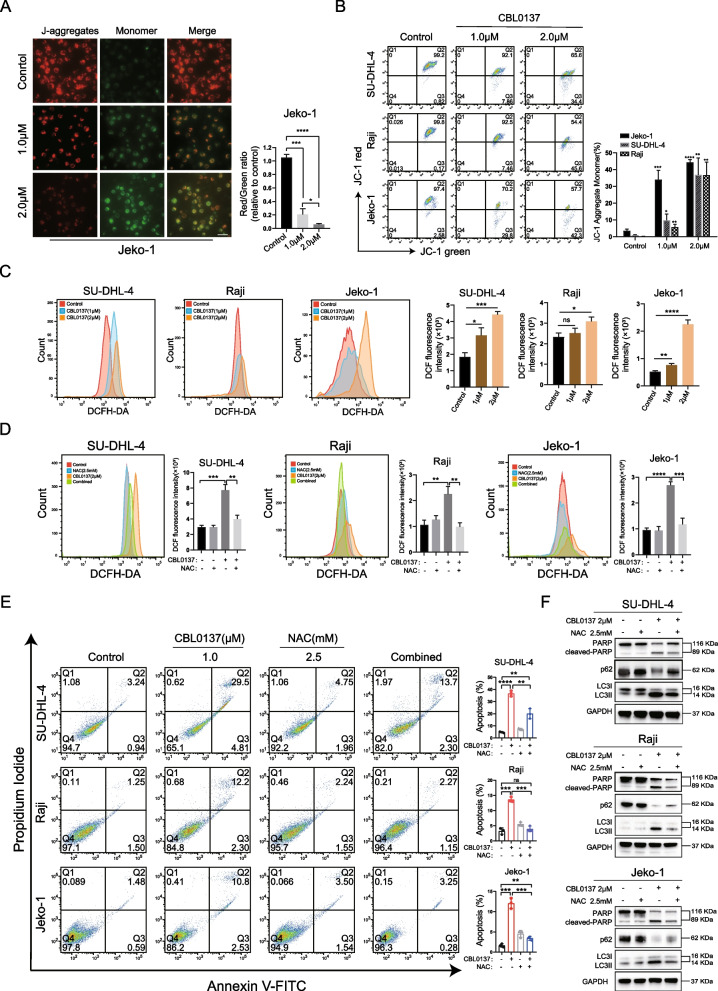
CBL0137-induced apoptosis and autophagy are dependent on mitochondrial ROS generation. **A** Jeko-1 cells treated with 1.0 μM or 2.0 μM CBL0137 were stained with the fluorescent mitochondrial probe JC-1, and the mitochondrial membrane potential (MMP) of the cells was observed by fluorescence microscope (40×, magnification). The quantitative results were analyzed. **B** The MMP stained with JC-1 probe was measured by flow cytometry. The histogram showed the percentage of JC-1 aggregate monomer. The data were expressed as mean ± SD (n = 3). **C** B-NHL cells were treated with different concentrations of CBL0137 for 24 h and then incubated with 10 μM DCFH-DA at 37 °C in the dark for 20 min, the fluorescent intensity was detected by flow cytometry. The mean fluorescence intensity of ROS was shown in histograms. The data were expressed as mean ± SD (n = 3). **D** B-NHL cells were preincubated with 2.5 mM NAC for 2 h and then treated with 2.0 μM CBL0137 for 24 h. The fluorescence intensity was detected by flow cytometry. **E** B-NHL cells were preincubated with 2.5 mM NAC for 2 h and then treated with 1.0 μM CBL0137 for 24 h. The changes in cell apoptosis rates were measured by flow cytometry. **F** In the presence or absence of NAC preincubation, the changes of apoptosis and autophagy-related proteins expression in B-NHL cells after CBL0137 treatment were detected by western blotting. *P < 0.05, **P < 0.01, ***P < 0.001, ****P < 0.0001

Aberrant production of ROS is usually involved in apoptosis and/or autophagy initiation [35, 36]. Mitochondria are the major cellular sites for ROS production [37]. First, we measured the mitochondrial membrane potential (MMP) with the fluorescence probe JC-1 and observed a decrease in red fluorescence (aggregated JC-1) and an increase in green fluorescence (monomeric JC-1). Flow cytometry further demonstrated a sharp decrease in the red/green fluorescence ratio (Fig. 3A, B). This finding led us to hypothesize that CBL0137 might trigger ROS release from mitochondria. Next, we used the fluorescent probe DCFH-DA to measure ROS levels by flow cytometry. As shown in Fig. 3C, CBL0137 dose-dependently increased ROS generation in SU-DHL-4 cells compared with untreated cells. Increased ROS generation was observed in Jeko-1 cells after 2.0 μM CBL0137 treatment but not significantly in Raji cells. Moreover, CBL0137-induced ROS overproduction was effectively blocked by pretreatment of the cells with the antioxidant agent N-acetylcysteine (NAC) (Fig. 3D). Subsequently, we analyzed the role of ROS in CBL0137-induced apoptosis and autophagy in the presence of NAC. Flow cytometry analysis showed that NAC significantly attenuated the CBL0137-induced apoptosis of B-NHL cells (Fig. 3E). Western blotting also demonstrated that NAC significantly inhibited CBL0137-induced activation of cleaved PARP. Furthermore, NAC markedly suppressed the increased expression of LC3II and consumption of p62 induced by CBL0137 (Fig. 3F). The above results indicated that ROS plays an important role in CBL0137-induced apoptosis and autophagy in B-NHL cells.

### CBL0137 induces apoptosis and autophagy in B-NHL cells via the ROS-mediated inhibition of the PI3K/Akt/mTOR and MAPK signaling pathways

**Fig. 4:**
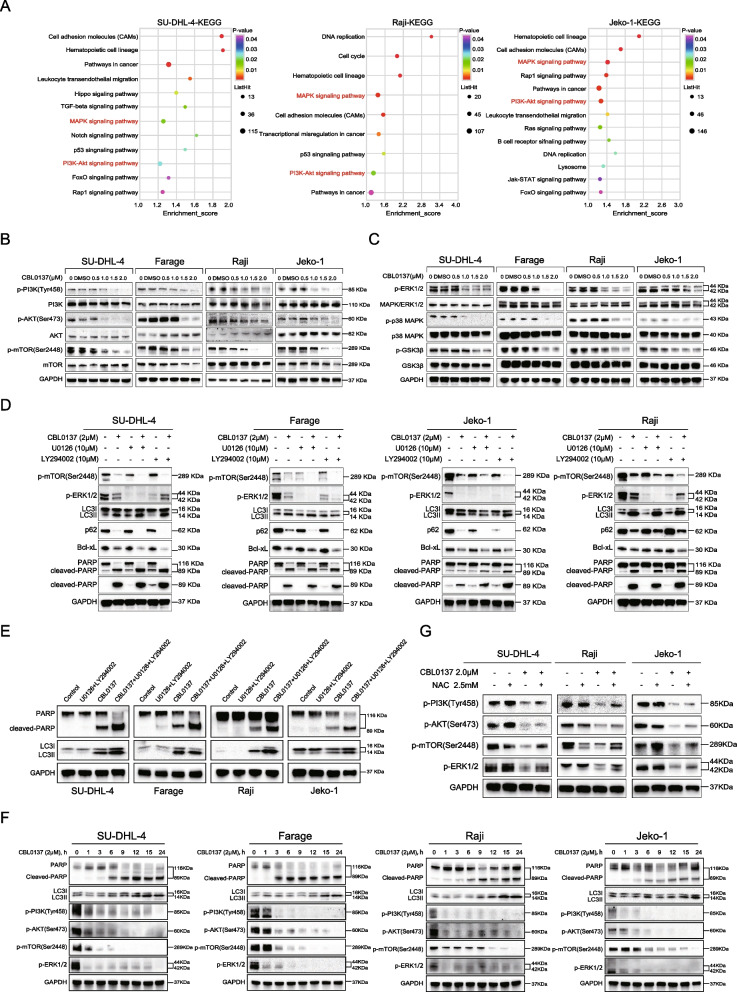
Effect of the PI3K/Akt/mTOR and MAPK signaling pathways on CBL0137-induced apoptosis and autophagy. **A** Singal pathway enrichment analysis of CBL0137 2.0 μM treated and untreated groups by RNA-seq in SU-DHL-4, Raji, and Jeko-1 cells (*P* < 0.05). **B** B-NHL cells were incubated with CBL0137 (0.5–2.0 μM) for 24 h, after which levels of PI3K, AKT, mTOR, and their phosphorylated forms were determined by western blotting. **C** Western blotting was used to detect the expression changes of related downstream molecules in the MAPK signaling pathway after CBL0137 (0.5–2.0 μM) treatment, including ERK1/2, p38 MAPK, p-p38 MAPK, GSK3β, and their phosphorylated forms. **D** B-NHL cells were pretreated with 10 μM U0126 or 10 μM LY294002 for 2 h followed by exposed to 2.0 μM CBL0137 for 24 h, respectively. Western blotting was used to detect the expression of apoptotic and autophagic marker proteins, and GAPDH was used as an internal control. **E** The expression level of cleaved PARP and the conversion of LC3 were analyzed by western blotting when 10 μM U0126 and 10 μM LY294002 were used together. **F** B-NHL cells were treated with 2.0 μM CBL0137 from 0 to 24 h. Western blotting was used to analyze the expression of key molecules involved in apoptosis and autophagy, as well as changes in the phosphorylation status of PI3K, Akt, mTOR, and ERK1/2. **G** B-NHL cells were treated with CBL0137 or in combination with NAC. The expression of p-PI3K, p-AKT, p-mTOR, and p-ERK1/2 was detected by western blotting in B-NHL cells

To further explore the potential mechanisms involved in the antitumor effects of CBL0137 in B-NHL, we applied RNA-seq to assess the transcriptional changes mediated by CBL0137. A total of 2283 and 927 genes were upregulated and downregulated in SU-DHL-4 cells, 1987 and 1234 genes were upregulated and downregulated in Raji cells, and 2405 and 1924 genes were upregulated and downregulated in Jeko-1 cells, respectively (Additional file 1: Fig. S4). Interestingly, KEGG pathway enrichment analysis showed that the PI3K/Akt and MAPK signaling pathways were significantly enriched (Fig. 4A). Previous studies have shown that the PI3K/Akt and MAPK signaling pathways play a pivotal role in mediating the crosstalk between tumor cell apoptosis and autophagy [38–42]. Therefore, we further verified the protein expression levels associated with these two pathways by western blotting (Fig. 4B, C) and found decreased levels of the related proteins. Together, these data suggested that CBL0137 inhibited the activation of the PI3K/Akt/mTOR and MAPK signaling pathways.

To investigate whether CBL0137-induced apoptosis and autophagy are associated with the PI3K/Akt/mTOR and MAPK signaling pathways, we exposed cells to LY294002 (a PI3K/Akt inhibitor) and U0126 (an ERK inhibitor) for 2 h and then treated them with or without 2.0 μM CBL0137 for 24 h. As shown in Fig. 4D, U0126 alone or in combination with CBL0137 significantly inhibited the phosphorylation level of ERK1/2 in B-NHL cells, indicating that U0126 could effectively target ERK1/2 expression in B-NHL cells. LY294002 strongly blocked mTOR phosphorylation in the presence of CBL0137. Meanwhile, we observed that in the presence of both a pathway inhibitor (U0126 or LY294002) and CBL0137, compared with CBL0137 alone, the expression of cleaved PARP increased more significantly in Jeko-1 cells, Bcl-xL expression decreased more significantly in SU-DHL-4 and Farage cells, and p62 consumption and LC3 conversion were more significantly in SU-DHL-4, Farage, and Jeko-1 cells. Based on these results, we further investigated the changes in apoptosis and autophagy of B-NHL cells after simultaneously blocking PI3K and MAPK pathways. Surprisingly, in all four B-NHL cell lines, we observed that CBL0137 combined with pathways double inhibition induced more significant apoptosis and autophagy than CBL0137 alone (Fig. 4E). In addition, to further elucidate the order in which the molecular effects occur, we conducted detailed time-course experiments from 0 to 24 h. Our results showed that a significant reduction in ERK1/2 phosphorylation was observed as early as 1 h during CBL0137 treatment. The phosphorylation status of PI3K decreased from 1 h after treatment, and the phosphorylation status of its downstream molecules Akt and mTOR decreased almost simultaneously or sequentially. Importantly, obvious apoptosis-specific PARP cleavage was not observed until 3 h of CBL0137 treatment, followed by induction of the conversion of LC3I to autophagosome-associated LC3II from 12 to 24 h (Fig. 4F). These results indicate that CBL0137-induced inhibition of the PI3K/Akt/mTOR and MAPK pathways occurs before apoptosis and autophagy. In conclusion, CBL0137-induced apoptosis and autophagy of B-NHL cells are mediated by inhibiting the PI3K/Akt/mTOR and MAPK signaling pathways.

Next, we evaluated the effects of ROS generation on the PI3K/Akt/mTOR and MAPK pathways. The results showed that compared with CBL0137 treatment alone, the combined treatment with CBL0137 and NAC increased the phosphorylation of mTOR in SU-DHL-4 cell line, reversing the phosphorylation inhibitory effect induced by CBL0137 treatment alone. The reversal of p-ERK1/2 was observed in all three B-NHL cell lines (Fig. 4G). In summary, CBL0137 induces apoptosis and autophagy in B-NHL cells through the ROS-mediated inhibition of the PI3K/Akt/mTOR and MAPK signaling pathways.

### CBL0137 targets NOTCH signaling in B-NHL cells

Previous studies have reported that the FACT inhibitor CBL0137 mediates small-cell lung cancer (SCLC) cell death by targeting NOTCH1 [16]. The RNA-seq results of the three B-NHL cell lines treated with CBL0137 showed that only SU-DHL-4 cells were significantly enriched in the NOTCH pathway (Fig. 4A). Many genes associated with the NOTCH signaling pathway were upregulated, such as NOTCH1, HES1, and p21 (Additional file 1: Fig. S5A).

Furthermore, western blotting showed that NOTCH1 was activated and that the expression of SP3 (negative regulator) decreased gradually with CBL0137 treatment (Additional file 1: Fig. S5B). Subsequently, the qRT‒PCR results showed that the mRNA expression levels of *NOTCH1*, *HEY1*, *HES1*, *p21*, and *PUMA* in four B-NHL cell lines were increased to varying degrees after treatment with CBL0137 (Additional file 1: Fig. S5C–E). These results indicate that CBL0137 could effectively target NOTCH signaling molecules in B-NHL and significantly activate NOTCH1 expression.

### CBL0137 in combination with rituximab enhances B-NHL cell death by promoting apoptosis and autophagy

**Fig. 5:**
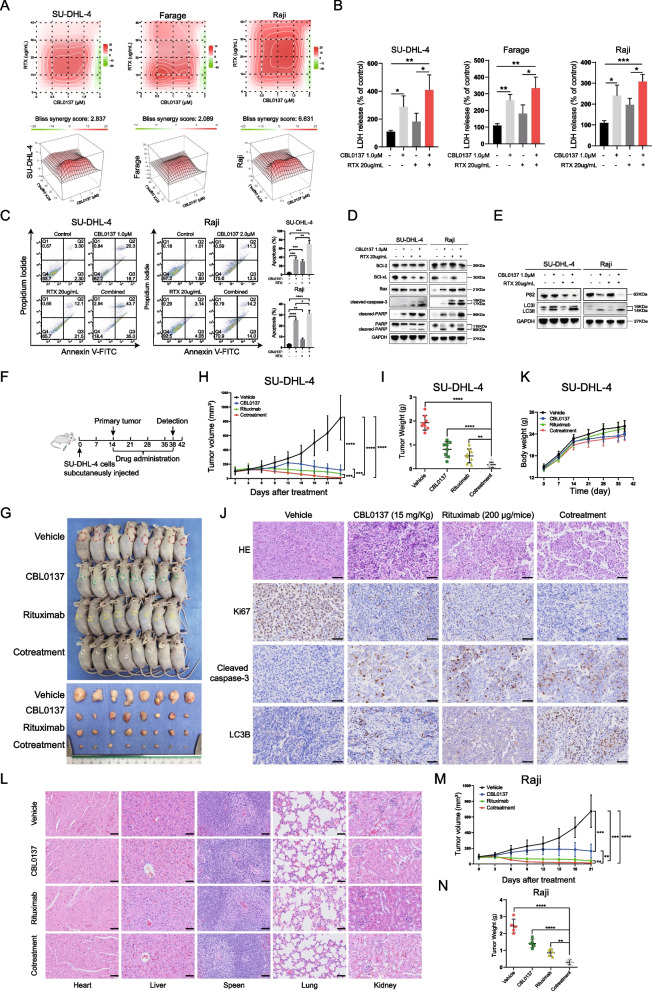
The combination of CBL0137 and rituximab exerted enhanced effects on B-NHL tumors in vitro and in vivo. **A** The synergy scores of CBL0137 and rituximab were calculated using the Bliss analysis. The concentration gradients of CBL0137 were 0.5, 1.0, 1.5, and 2.0 μM, and the concentration gradients of rituximab were 10, 20, 30, and 40 μg/mL. The combination responses of the two drugs were observed through the dose–response matrix. The 2D and 3D synergy maps above and below highlighted synergistic and antagonistic dose regions in red and green color respectively and showed the summary synergy scores of two drug combinations in three B-NHL cell lines. **B** LDH release assay of B-NHL cells treated with the indicated concentration of CBL0137 or rituximab and the combination of the two drugs for 24 h. **C** The Annexin V-FITC/PI staining results were shown on the left, which was that CBL0137 combined with rituximab has enhanced apoptotic effects on SU-DHL-4 and Raji cells. Statistical results of apoptosis assay were shown on the right, representing three independent experiments. (*P < 0.05, **P < 0.01, ***P < 0.001, Student’s t-test). **D**, **E** Representative western blotting results of B-NHL cells treated with the single or combination treatment for 24 h to determine changes in apoptosis-related proteins **D** and autophagy-related proteins **E** expression. **F** Experimental protocol of xenograft model of DLBCL. **G** SU-DHL-4 cells were subcutaneously implanted into male nude mice, and the mice were sacrificed after the treatment. The mice and their corresponding xenograft tumor specimens were photographed with a high-definition digital camera. **H** Tumor volume was measured every three days and tumor growth curves were plotted. ***P < 0.001, ****P < 0.0001. **I** Tumor weight in each group was measured and presented at the end of treatment. **P < 0.01, ***P < 0.001, ****P < 0.0001. **J** The body weight of mice in four groups (n = 8) was recorded weekly. **K** Histological examination of vital organs was performed by H&E staining. Scale bars = 50 μm. **L** Representative immunohistochemical images of Ki67, cleaved caspase-3, and LC3B were performed in the four groups of tumor samples. Scale bars = 50 μm. **M**, **N** Establishment of BL xenograft tumors. Raji cells were subcutaneously implanted into female nude mice, and the mice were sacrificed after the treatment. **M** Quantified results of tumor volume. **N** Quantified results of tumor weight

Rituximab is usually used to treat B-NHL patients either alone or in combination with chemotherapy as part of initial therapy or a second-line regimen. It has been reported that rituximab induces apoptosis in lymphoma cells by inhibiting various antiapoptotic constitutively activated signaling pathways, such as NF-κB, p38 MAPK, ERK1/2, AKT, Bcl-2, and Bcl-xL [43]. Therefore, to determine whether CBL0137 increases the sensitivity of B-NHL to rituximab, we treated B-NHL cells with 0.5, 1.0, 1.5, and 2.0 μM CBL0137 alone or in combination with 10, 20, 30, 40 μg/mL rituximab for 24 h. The overall conclusions about synergy, additivity, or antagonism for a drug combination between CBL0137 and rituximab are based on Bliss independence models (Fig. 5A). The CBL0137/rituximab drug pair yields summary synergy scores of 2.873 (SU-DHL-4), 2.089 (Farage), and 6.631 (Raji), indicating an overall additive effect on inhibiting B-NHL cell growth. The synergy score is from − 10 to 10: the interaction between two drugs is considered additive [44]. A cytotoxicity assay was performed to further verify the enhancement effect of the combination of the two drugs on tumor cell death in vitro. Increased LDH release was observed by the LDH release assay in B-NHL cells treated with CBL0137 or rituximab, and LDH release was more significant in the combination group (Fig. 5B), indicating that the combination treatment significantly increased the cell death effect. The percentage of early and late apoptotic cells in the combination treatment group (78.70%) was significantly higher than that of either 1.0 μM CBL0137 (37.00%) or rituximab (33.60%) in SU-DHL-4 cells (Fig. 5C). Further western blotting showed that cleaved caspase-3, cleaved-PARP, and Bax expression levels were higher in the combination groups than in the monotherapy groups, and the expression levels of Bcl-2 and Bcl-xL were decreased to varying degrees (Fig. 5D). Evidence suggests that rituximab-containing antibody‒drug conjugates (ADCs) induce effective antitumor efficacy by activating autophagy in ADC-based B-NHL therapy [45]. Our results found that although LC3 transformation was not obvious in the rituximab-treated groups, a more significant accumulation of LC3II was observed in the combination groups than in the CBL0137-treated groups. It was also proven that p62 consumption was more significant in the combination groups (Fig. 5E). All the above results suggest that CBL0137 combined with rituximab exerts a more significant antitumor effect in B-NHL cells by enhancing apoptosis and autophagy.

### CBL0137 and rituximab have enhanced antitumor effects in B-NHL models

To further validate the antitumor effects in vivo, two tumor-bearing xenografts of B-NHL subtypes were constructed (Fig. 5F). We found that treatment with CBL0137 or rituximab alone significantly reduced DLBCL tumor volumes and tumor weight compared with those of the vehicle group, but the combination with both CBL0137 and rituximab showed a maximum inhibitory effect on tumor growth (Fig. 5G–I). To further elucidate the mechanisms of the antitumor effects in vivo, we performed immunohistochemical analyses of tumor tissues. As shown in Fig. 5J, compared with the other groups, the cotreatment group demonstrated an increase in the tumor tissue necrotic area, as well as the most significantly decreased Ki67 expression level and the most significant increase in cleaved caspase-3 and LC3B expression. During the whole experiment, the body weight of the mice increased steadily in each group, and no significant difference was observed (Fig. 5K). H&E staining showed no evident damage to the major organs (heart, liver, spleen, lung, and kidney) of mice treated with CBL0137 and/or rituximab (Fig. 5L). On the other hand, the established BL model obtained similar results (Fig. 5M, N and Additional file 1: Fig. S6). Thus, CBL0137 combined with rituximab exhibited significant enhancement of the antitumor effects by inducing apoptosis and autophagy in vivo.

## Discussion

Chemoimmunotherapy is the standard for first- and second-line treatment in aggressive B-NHL patients. High-dose chemotherapy plus ASCT did not improve outcomes in patients with high-risk aggressive B-cell lymphoma and showed significant toxicity, according to the results of a new phase 3 trial with 10 years of follow-up [46]. Therefore, there is an urgent need to identify safer and cancer-selective multimodal therapies for aggressive B-NHL patients. Herein, we report on a novel combination of the anticancer drug CBL0137 and rituximab, focusing on the unique antitumor efficacy and mechanism of CBL0137 in multiple types of B-NHL cells.

CBL0137, as a second-generation curaxin, has been reported to have antitumor activity in many types of cancer, with high efficacy and minimal side effects [47, 48]. This study is the first to report the efficacy of CBL0137 in lymphoma and provides preclinical evidence of a novel combinatorial therapy with rituximab. CBL0137 exerts antitumor effects by targeting the FACT complex, disrupting DNA damage repair, and promoting chromatin depolymerization [49, 50]. The two subunits of the FACT complex, namely, SSRP1 and SUPT16H, interact and regulate each other [15]. FACT overexpression in multiple types of tumors is associated with poor prognosis [9, 11–13, 15]. Therefore, targeting FACT is an attractive target for cancer therapy. In this research, we demonstrated that FACT is a potential therapeutic target for B-NHL, and both SSRP1 and SUPT16H are significantly overexpressed in DLBCL tumor tissues and are closely related. Mechanistically, our data further showed that CBL0137 has tumor-suppressive effects in B-NHL cells by inhibiting the NF-κB pathway, activating the p53 pathway, and inducing chromatin trapping of FACT (Additional file 1: Fig. S1), which is the classical antitumor mechanism of CBL0137.

CBL0137 can also inhibit SCLC cell growth by activating NOTCH1 signaling [13]. Multiple studies have shown that changes in NOTCH signaling are closely related to the development of lymphoma. Targeting NOTCH signaling can not only improve lymphoma treatment but can also prevent lymphoma-to-myeloid tumor conversion [51–54]. It was pleasantly surprised to find in our study that CBL0137 induces NOTCH pathway activation and changes in transcription, proliferation, and cycle-related target genes in B-NHL cells (Additional file 1: Fig. S5). Therefore, we hypothesized that CBL0137 might mediate B-NHL cell death by targeting the NOTCH pathway and its target genes.

Dysregulation of the cell cycle and intense proliferation are essential characteristics of malignant tumor cells. We found a significant increase in the number of B-NHL cells in S phase after treatment with CBL0137. From a molecular perspective, CDKs and cyclins play an important role in controlling cell cycle progression [55]. Evidence suggests that cyclins could interact with the CDK family and sequentially induce upregulation and downregulation expression changes, thereby significantly increasing the percentage of cells in S phase [56–58]. Correspondingly, we found that cyclin A2, B1, and E1 were upregulated. In addition, cyclin D1, another indicator of S phase arrest, which is inhibited only in the S phase [59], was also consistent with our results. Moreover, we also found that the expression levels of cell cycle-related regulatory genes (cyclin A2, cyclin B1, cyclin D1, cyclin E1, CDK1, and CDK2) were regulated by upregulating the p53/p21 genes and reducing c-MYC. Collectively, our results confirmed that S phase arrest in B-NHL cells is mainly driven by a synergistic mechanism, which sequentially changes the expression of CDKs and cyclins by modulating the c-MYC/p53/p21 pathway (Fig. 1D, E).

Apoptosis and autophagy are the two main mechanisms leading to programmed cell death. Unlike apoptosis, the role of autophagy in cancer is complex [30, 60]. Under certain stress conditions, upregulation of autophagy may lead to cell death. Along with the selective degradation of cellular components, autophagy also provides a cell-survival pathway by recycling selective intracellular organelles and proteins [61]. Previous reports have shown that apoptosis and autophagy can collectively trigger cell death through synergistic, complementary, or alternative mechanisms [31]. Inhibition of autophagy could enhance apoptosis in oral cancer cells [62], and autophagy promotes apoptosis in acute myeloid leukemia cells [23]. In our present study, we demonstrated that CBL0137 causes cell death mainly through the induction of apoptosis, accompanied by the generation of autophagy. Further inhibition of autophagy promoted CBL0137-induced cell death (Fig. 2D–G), suggesting that CBL0137 promotes a survival feedback loop by inducing autophagy. Moreover, some evidence has demonstrated that ROS can induce apoptosis and/or activate autophagy, depending on the cellular components [35, 36]. It is worth noting in our study that CBL0137 increased the generation of ROS. In addition, the ROS inhibitor NAC significantly inhibited apoptosis and autophagy, further suggesting that CBL0137 induced ROS generation, which contributed to cell death (Fig. 3). Furthermore, apoptosis and autophagy share many signaling pathways, such as the PI3K/Akt/mTOR and MAPK signaling pathways [39, 40, 42]. However, the molecular mechanisms of ROS-mediated apoptosis and autophagy with CBL0137 treatment are still not clear. Therefore, we used pathway inhibitors, such as LY294002 and U0126, to further confirm the related signaling pathways (Fig. 4). Hence, these results indicated that CBL0137 induces ROS-mediated apoptosis and autophagy by triggering the PI3K/Akt/mTOR and MAPK signaling pathways.

Based on our results, we found that CBL0137 exerts multiple antitumor effects in aggressive B-NHL tumors. Mechanistically, we proposed a model in which CBL0137 could induce cell cycle arrest by regulating the c-MYC/p53/p21 pathway and inhibiting NF-κB transcription of FACT, resulting in direct inhibition of cancer cell growth. Most importantly, CBL0137 could also induce cell apoptosis and protective autophagy through caspase-dependent pathways and depended on the ROS-mediated PI3K/Akt/mTOR and MAPK pathways. A parallel pathway boosts an enhanced response against B-NHL tumor progression. These anticancer mechanisms are interconnected, ultimately resulting in tumor cell death. Our study found that CBL0137 could induce cell cycle arrest by inhibiting c-MYC and FACT expression. Consistently, a study indicated that MYC expression was significantly suppressed after FACT subunit knockout to maintain the latency of Epstein‒Barr virus (EBV)-infected Burkitt lymphoma (BL) [63]. When FACT was silenced, c-MYC expression was reduced, and tumor cells were prevented from re-entering the cell cycle [64]. Moreover, the use of CBL0137 inhibited the FACT/MYC positive feedback loop and increased apoptosis in neuroblastoma tumors [11]. These results strongly suggest that FACT-driven MYC expression is a druggable target in malignant tumors. In addition, CBL0137 induces apoptosis and autophagy in human B-NHL cells via the ROS-mediated PI3K/Akt/mTOR and MAPK signaling pathways, which is the main mechanism of its antitumor effect and the first reported mechanism thus far. Inhibition of autophagy with chloroquine enhanced the ability of tumor cells to undergo apoptosis in the MYC-induced model of lymphoma [65], and this protective autophagy ability is also reflected in our results. Another study proposed that c-MYC inhibition sensitizes acute lymphoblastic leukemia (ALL) to chemotherapy by altering caspase-3-dependent apoptosis and autophagy by targeting the PI3K pathway [66]. Recently, some researchers have also proposed that inhibition of FACT subunits by SSRP1 silencing enhanced apoptosis of cancer cells upon cisplatin treatment [67–70]. Our study also found that NOTCH1 was activated as a tumor suppressor after treatment with CBL0137. It is now widely recognized that the MYC transcription factor is a dominant oncogenic driver in a majority of human T-ALL and is often activated downstream of NOTCH1 [71], and targeting the NOTCH/MYC pathway can lead to cell death by increasing the apoptosis of tumor cells. In summary, the anticancer mechanisms of CBL0137 that we have shown are interrelated.

To our knowledge, we are the first group to demonstrate that CBL0137 enhances rituximab antitumor activity in vitro and in vivo (Fig. 5). Based on in vitro data, the mouse model further strongly proved the significant efficacy of the cotreatment therapy. Importantly, using in vivo xenograft models, we demonstrated that while rituximab or CBL0137 treatment alone had a moderate effect on the suppression of B-NHL, the combination of rituximab and CBL0137 significantly suppressed B-NHL growth in vivo. Further immunohistochemical assays showed that the expression levels of cleaved caspase-3 and LC3 were significantly upregulated after CBL0137 treatment, providing evidence that CBL0137 inhibited B-NHL tumor growth by cell apoptosis and autophagy in a xenograft model.

## Conclusions

In conclusion, we report that CBL0137 induces apoptosis and autophagy in human B-NHL cells via the ROS-mediated PI3K/Akt/mTOR and MAPK signaling pathways and reveals a significant enhancing effect with the combination of rituximab. This work provides credible preclinical evidence for pending and ongoing clinical trials of CBL0137. Furthermore, this new combination therapy may provide more options for the treatment of aggressive B-NHL patients in the future.

## Supplementary Information

**Additional file 1:** Supplementary Materials and Methods. **Fig. S1**. CBL0137 exerts antitumor activity by inhibiting FACT function and regulating p53 and NF-κB activity. **Fig. S2**. CBL0137 induces S phase cell cycle arrest and apoptosis in B-NHL cells. **Fig. S3**. CBL0137 induces autophagy in human B-NHL cells. **Fig. S4**. Differentially expressed genes (DEGs) were analyzed in SU-DHL-4, Raji, and Jeko-1 cell. **Fig. S5**. CBL0137 targets NOTCH signaling in B-NHL cells. **Fig. S6**. CBL0137 showed enhanced effects with rituximab in inhibiting the growth of BL xenograft tumors in vivo. **Table S1**. Source and identifier of reagents used in this study. **Table S2**. Source and identifier of antibodies used in this study. **Table S3**. The primers sequences for qRT-PCR in this study.

## Data Availability

The RNA-seq datasets supporting the conclusions of this article are available in the Gene Expression Omnibus (GEO) with Accession No. GSE208679 (http://www.ncbi.nlm.nih.gov/geo/).

## Abbreviations

B-NHL: B-cell non-Hodgkin’s lymphoma
DLBCL: Diffuse large B-cell lymphoma
BL: Burkitt lymphoma
MCL: Mantle cell lymphoma
ROS: Reactive oxygen species
SSRP1: Structure-specific recognition protein 1
SPT16: Suppressor of Ty-16
FACT: Facilitates chromatin transcription
TEM: Transmission electron microscopy
CDK: Cyclin-dependent kinase
MMP: Mitochondria membrane potential
