# Supplementary material for: The FACT-targeted drug CBL0137 enhances the effects of rituximab to inhibit B-cell non-Hodgkin’s lymphoma tumor growth by promoting apoptosis and autophagy
Source: Cell Commun Signal. 2023 Jan 23;21:16. doi: 10.1186/s12964-022-01031-x (PMC9869543; doi:10.1186/s12964-022-01031-x)
Supplement: Supplementary file 2 — Additional file 1: Supplementary Materials and Methods. Fig. S1. CBL0137 exerts antitumor activity by inhibiting FACT function and regulating p53 and NF-κB activity. Fig. S2. CBL0137 induces S phase cell cycle arrest and apoptosis in B-NHL cells. Fig. S3. CBL0137 induces autophagy in human B-NHL cells. Fig. S4. Differentially expressed genes (DEGs) were analyzed in SU-DHL-4, Raji, and Jeko-1 cell. Fig. S5. CBL0137 targets NOTCH signaling in B-NHL cells. Fig. S6. CBL0137 showed enhanced effects with rituximab in inhibiting the growth of BL xenograft tumors in vivo. Table S1. Source and identifier of reagents used in this study. Table S2. Source and identifier of antibodies used in this study. Table S3. The primers sequences for qRT-PCR in this study. [file 12964_2022_1031_MOESM2_ESM.pdf]

Supplementary Materials for

The FACT-targeted drug CBL0137 enhances the effects of rituximab to inhibit B-cell non-Hodgkin's lymphoma tumor growth by promoting apoptosis and autophagy

Yan Lv[1,4], Yuxin Du[1,4*], Kening Li[2], Xiao Ma[3], Juan Wang[1], Tongde Du[1], Yuxin Ma[1], Yue Teng[1], Weiyan Tang[1], Rong Ma[1], Jianqiu Wu[1], Jianzhong Wu[1], Jifeng Feng[1*]

*Correspondence: Jifeng Feng (jifengfeng2021@gmail.com) and Yuxin Du (yuxindu0408@gmail.com).

The PDF file includes:

- Supplementary Materials and Methods
- Fig. S1. CBL0137 exerts antitumor activity by inhibiting FACT function and regulating p53 and NF-κB activity.
- Fig. S2. CBL0137 induces S phase cell cycle arrest and apoptosis in B-NHL cells.
- Fig. S3. CBL0137 induces autophagy in human B-NHL cells.
- Fig. S4. Differentially expressed genes (DEGs) were analyzed in SU-DHL-4, Raji, and Jeko-1 cell.
- Fig. S5. CBL0137 targets NOTCH signaling in B-NHL cells.
- Fig. S6. CBL0137 showed enhanced effects with rituximab in inhibiting the growth of BL xenograft tumors *in vivo*.
- Supplementary figure legends
- Table S1. Source and identifier of reagents used in this study.
- Table S2. Source and identifier of antibodies used in this study.
- Table S3. The primers sequences for qRT-PCR in this study.

Supplementary Materials and Methods

Reagents and antibodies

Reagents and antibodies are detailed in **Table S1 and Table S2**. For *in vitro* experiments, a stock solution of CBL0137, NAC, CQ, U0126, and LY294002 were prepared in 100% DMSO, stored at -80 °C, and further diluted in RPMI-1640 to the indicated concentrations. Vehicle-treated controls contained a final *in vitro* DMSO concentration of 0.1%. Furthermore, Rituximab was diluted in normal saline, while CBL0137 was prepared in double-distilled water, for use in the *in vivo* experiments.

Cell proliferation assay

SU-DHL-4, Farage, Raji, and Jeko-1 cells (1~2 × 10^4 cells/well) were seeded in 96-well plates and then treated with CBL0137 at various concentrations for 24 h, 48 h, or 72h. Totally 10% CCK-8 solution was added to each well and incubated for 1 h. The absorbance was measured at 450nm with a microplate reader. The mean value of optical density of four wells was taken to calculate cell viability by the following formula: Cell viability (%) = $(OD_{(treatment\ group)}-OD_{(blank\ group)}) / (OD_{(control\ group)}-OD_{(blank\ group)}) \times 100\%$. Thus, growth inhibition was calculated by the following formula: (100%-cell viability). The IC_{50} values were calculated using GraphPad Prism 8.0 software.

Formal synergy assays of CBL0137 and rituximab were performed using Bliss analysis. The concentration gradients of CBL0137 were 0.5, 1.0, 1.5, and 2.0 μM, and the concentration gradients of rituximab were 10, 20, 30, and 40 μg/mL. The combination responses of the two drugs were observed through the dose-response matrix. Interactive analysis and visualization of two-drug combination screening data using SynergyFinder

application [1]. The summary synergy scores were used to evaluate the interaction between the two drugs. Less than -10 represents antagonistic effect, from -10 to 10 represents additive effect, and larger than 10 represents synergistic effect.

Analysis of reactive oxygen species (ROS) and mitochondrial membrane (Δψm) levels by flow cytometry in B-NHL cells

Intracellular ROS was determined by the Reactive Oxygen Species Assay Kit (Solarbio, China). Cells were seeded in six-well plates overnight and exposed to indicated concentrations of CBL0137, with or without NAC (2.5 mM) for 24 h. Cells were collected and resuspended in a serum-free medium containing 10 μM DCFH-DA ROS probes. DCF fluorescence intensity was then detected by flow cytometry.

The mitochondrial membrane potential (Δψm) was determined using a JC-1 Staining Dye Assay Kit (Solarbio, China) according to the manufacturer's instructions. Briefly, cells after treatment with CBL0137 (1.0 μM or 2.0 μM) for 24 h, stained with JC-1 working solution at 37 °C in the dark for 20 min, washed twice with JC-1 buffer solution, then resuspended again, and subsequently observed by fluorescence microscope and analyzed by flow cytometry. The decrease of the red/green fluorescence ratio indicated that mitochondrial membrane potential decreased, which could be used as an indicator of early apoptosis.

Preparation of cell extracts and western blotting analysis

Total proteins from SU-DHL-4, Farage, Raji, and Jeko-1 cells after the drug treatment were lysed in ice-cold RIPA buffer (Thermo Fisher Scientific, USA) with protease and phosphorylase inhibitors (NCM Biotech, China) for 30min. Lysates were centrifuged at

12000rpm for 20min at 4 °C, and then collected the supernatant, which was the total protein. Cytoplasmic and nuclear proteins were prepared using a Nuclear and Cytoplasmic Protein Extraction Kit (Beyotime, China). The concentration of protein was quantified using the BCA Protein Assay Kit (Beyotime, China). Equal amounts of protein extracts were separated by 4%~20% gradient SDS-PAGE (GenScript ProBio, USA) and transferred to nitrocellulose membranes (Cytiva, Germany). The membranes were blocked in 5% non-fat milk at room temperature for 1 h and then incubated with specific primary antibodies at 4 °C overnight, and then incubated with horseradish peroxidase (HPR)-conjugated secondary antibodies (CST, USA) for 1 h. The protein bands were visualized by enhanced chemiluminescence (Millipore, USA) and analyzed using ImageJ software.

RNA extraction and real-time quantitative polymerase chain reaction (PCR)

Total RNA was extracted using EZ-10 DNAaway RNA Mini-Preps Kit (Sangon Biotech, China). After quantification by spectrophotometry, the first-strand cDNA was synthesized from 1000 ng of total RNA using RNA Reverse PCR Kit (TaKaRa, Japan) and used as a template by 5-fold dilution. Three replicates were prepared for each group of samples. qRT-PCR was performed with the PowerUp SYBR Green Master Mix (Thermo Fisher Scientific, USA) according to the instructions. Relative gene expression was analyzed by the $2^{-\triangle\triangle Ct}$ method with GAPDH RNA as endogenous control. The primers sequences for qRT-PCR are as shown in **Table S3**.

RNA sequencing

RNA-seq was used to analyze gene expression profiles of SU-DHL-4, Raji, and Jeko-1 cells treated with CBL0137 (2.0 μM) for 24 h and without treatment. Three biological

replicates were performed for each sample. The quality and quantity of each RNA were measured using an Agilent 2100 Bioanalyzer (Agilent, Santa Clara, CA, USA). The RNA sequencing libraries were prepared using the TruSeq Stranded mRNA LTSample Prep Kit (Illumina, San Diego, CA, USA) according to the manufacturer's instructions. Then these libraries were sequenced by HiSeq 2500 and output 150 bp paired-end reads. Raw data were processed using Trimmomatic and obtain the clean reads. The clean reads were mapped to hg19 using hisat2. Fragments per kilobase per million reads sequenced (FPKM) and the read counts value of each transcript was calculated using bowtie 2 and express. Different expressed genes (DEGs) were identified using the DESeq (2012) functions estimateSizeFactors and nbinomTest.

Xenograft mice model of human B-NHL

All experiments involving animals were approved by the Institutional Animal Care and Use Committee of Nanjing Medical University. Female and male BALB/c nude mice (4 weeks old) purchased from GemPharmatech. The mice were housed in barrier facilities with a 12-hour light-dark cycle, with food and water available ad libitum. SU-DHL-4 cells (3 × 10^7 cells/150 μL serum-free 1640 medium) were subcutaneously inoculated into the flanks of male nude mice, and Raji cells (2 × 10^7 cells/150 μL serum-free 1640 medium) were subcutaneously inoculated into the flanks of female nude mice. After the development of 5 mm diameter tumors, mice were randomly assigned to four groups (SU-DHL-4 inoculated group, n = 8; Raji inoculated group, n = 5): (1) vehicle control group, injected intraperitoneally with 0.9% normal saline (NS); (2) CBL0137-treated group, injected intraperitoneally with CBL0137 (15 mg/kg); (3) rituximab-treated group, injected

intraperitoneally with rituximab (200 μg/mice); (4) Co-treatment group, injected intraperitoneally with CBL0137 (15 mg/kg) and rituximab (200 μg/mice). Mice were dosed every three days and the effect of treatment was monitored by measuring tumor size every three days. The tumor diameter was measured with a vernier caliper and the tumor volume was calculated using the following formula: 0.5 × length × $width^2$. The body weight of the mice was monitored weekly. Mice were sacrificed by cervical dislocation when the tumor reached 1,000 mm^3. Tumor xenografts were then excised, weighted, stored, and fixed.

To test the toxicity of CBL0137 and rituximab *in vivo*, mice in each group were randomly selected for dissection after the mice were killed, and their heart, liver, spleen, lung, and kidney were stained with hematoxylin and eosin (H&E) to observe whether there were obvious abnormalities. Meanwhile, H&E, Ki67, cleaved caspase-3, and LC3B staining were performed on the tumor xenografts according to the manufacturer's instructions.

References

1. Ianevski A, Giri AK, Aittokallio T. SynergyFinder 3.0: an interactive analysis and consensus interpretation of multi-drug synergies across multiple samples. Nucleic Acids Res. 2022.

Fig. S1

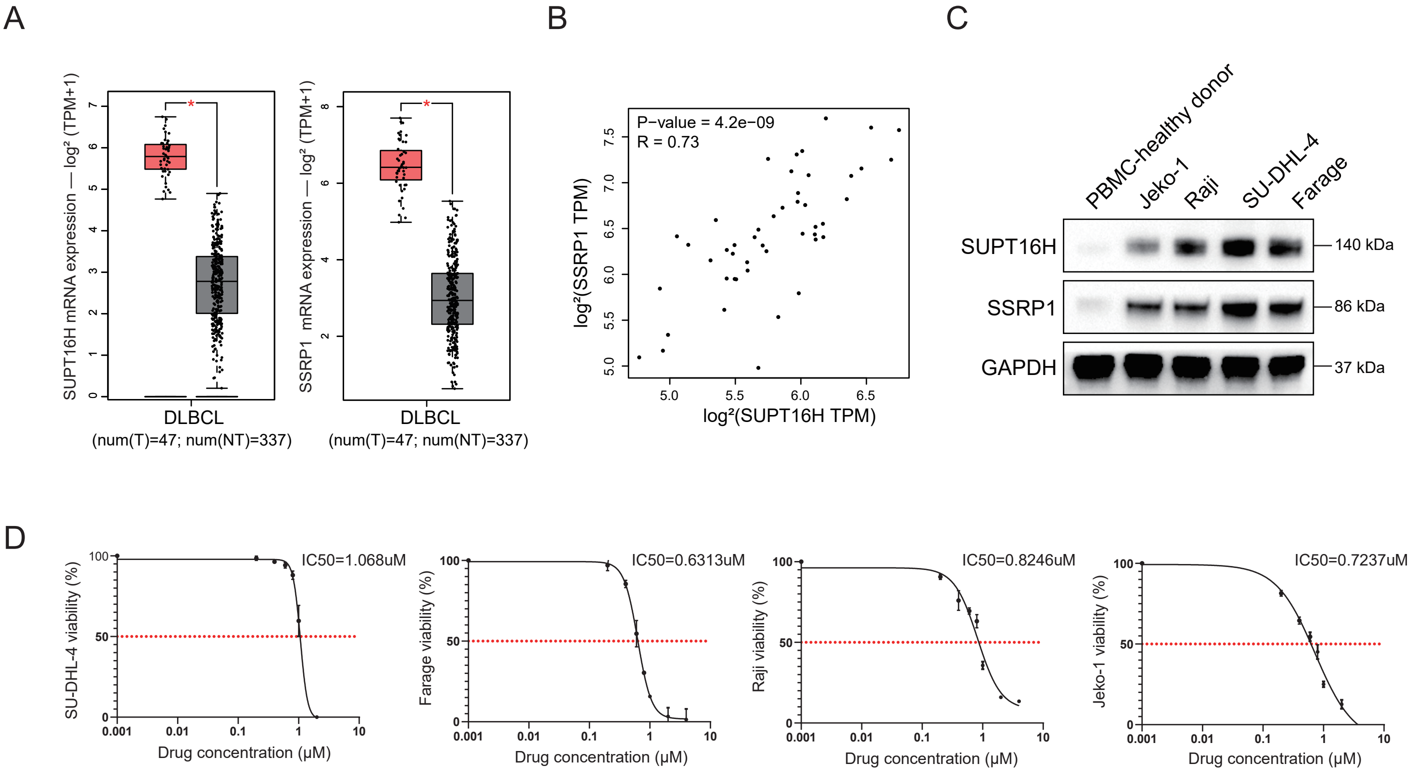

E

Table I. 24-hour IC50 (μM) of CBL-0137 in different B-NHL cells

Cell lines	IC50	95%CI
Raji	0.8246	0.7141 ~ 0.9753
Jeko-1	0.7237	0.6163 ~ 0.9088
SU-DHL-4	1.068	1.034 ~ 1.122
Farage	0.6313	0.6001~0.6632

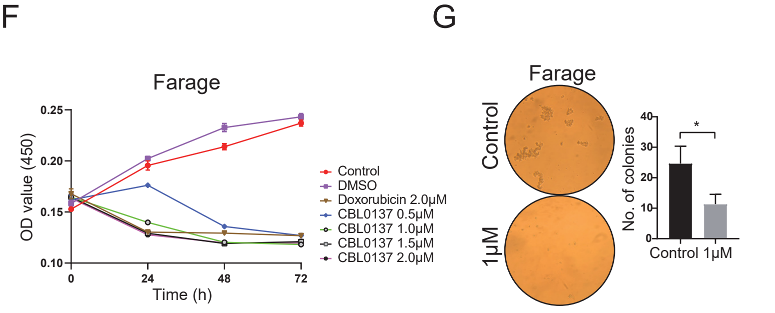

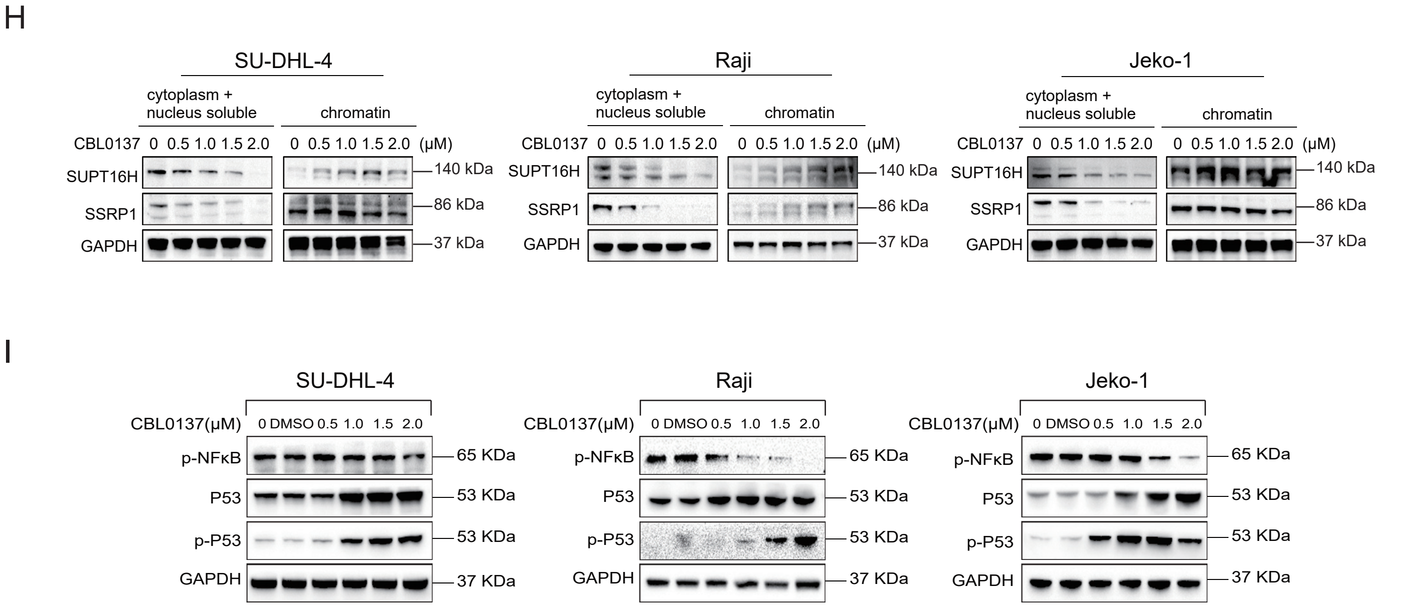

Fig. S2

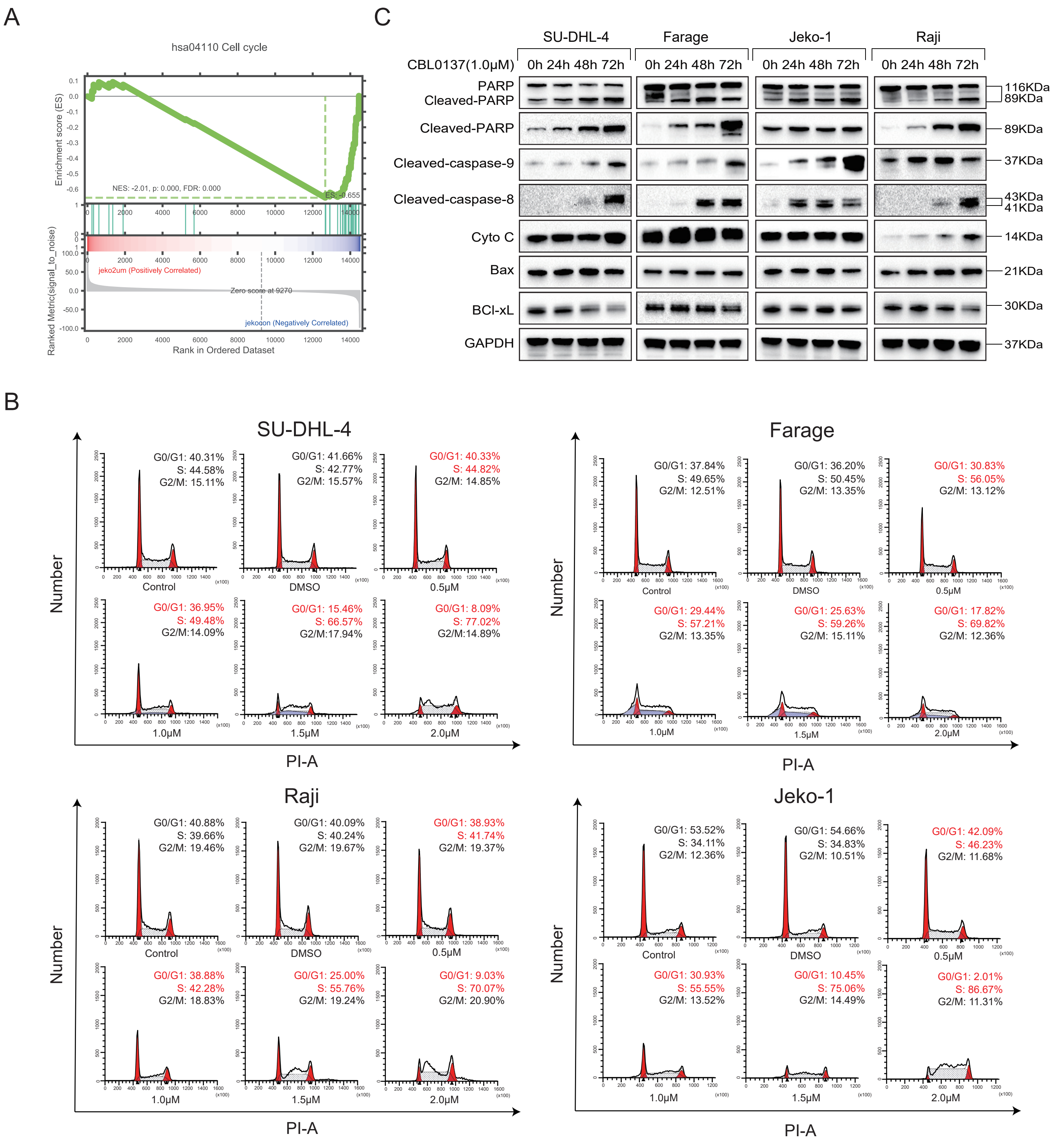

A
hsa04110 Cell cycle
Enrichment score (ES)
NES: -2.01, p: 0.000, FDR: 0.000
ES: -0.655
Ranked Metric(signal_to_noise)
jeko2um (Positively Correlated)
Zero score at 9270
jekocon (Negatively Correlated)
Rank in Ordered Dataset
C
SU-DHL-4
Farage
Jeko-1
Raji
CBL0137(1.0μM)
0h 24h 48h 72h
PARP
Cleaved-PARP
Cleaved-caspase-9
Cleaved-caspase-8
Cyto C
Bax
BCl-xL
GAPDH
116KDa
89KDa
37KDa
43KDa
41KDa
14KDa
21KDa
30KDa
B
SU-DHL-4
G0/G1: 40.31% S: 44.58% G2/M: 15.11%
Control
G0/G1: 41.66% S: 42.77% G2/M: 15.57%
DMSO
G0/G1: 40.33% S: 44.82% G2/M: 14.85%
0.5μM
G0/G1: 36.95% S: 49.48% G2/M:14.09%
1.0μM
G0/G1: 15.46% S: 66.57% G2/M:17.94%
1.5μM
G0/G1: 8.09% S: 77.02% G2/M:14.89%
2.0μM
Number
PI-A
Farage
G0/G1: 37.84% S: 49.65% G2/M: 12.51%
Control
G0/G1: 36.20% S: 50.45% G2/M: 13.35%
DMSO
G0/G1: 30.83% S: 56.05% G2/M: 13.12%
0.5μM
G0/G1: 29.44% S: 57.21% G2/M: 13.35%
1.0μM
G0/G1: 25.63% S: 59.26% G2/M: 15.11%
1.5μM
G0/G1: 17.82% S: 69.82% G2/M: 12.36%
2.0μM
Raji
G0/G1: 40.88% S: 39.66% G2/M: 19.46%
Control
G0/G1: 40.09% S: 40.24% G2/M: 19.67%
DMSO
G0/G1: 38.93% S: 41.74% G2/M: 19.37%
0.5μM
G0/G1: 38.88% S: 42.28% G2/M: 18.83%
1.0μM
G0/G1: 25.00% S: 55.76% G2/M: 19.24%
1.5μM
G0/G1: 9.03% S: 70.07% G2/M: 20.90%
2.0μM
Jeko-1
G0/G1: 53.52% S: 34.11% G2/M: 12.36%
Control
G0/G1: 54.66% S: 34.83% G2/M: 10.51%
DMSO
G0/G1: 42.09% S: 46.23% G2/M: 11.68%
0.5μM
G0/G1: 30.93% S: 55.55% G2/M: 13.52%
1.0μM
G0/G1: 10.45% S: 75.06% G2/M: 14.49%
1.5μM
G0/G1: 2.01% S: 86.67% G2/M: 11.31%
2.0μM

Fig. S3

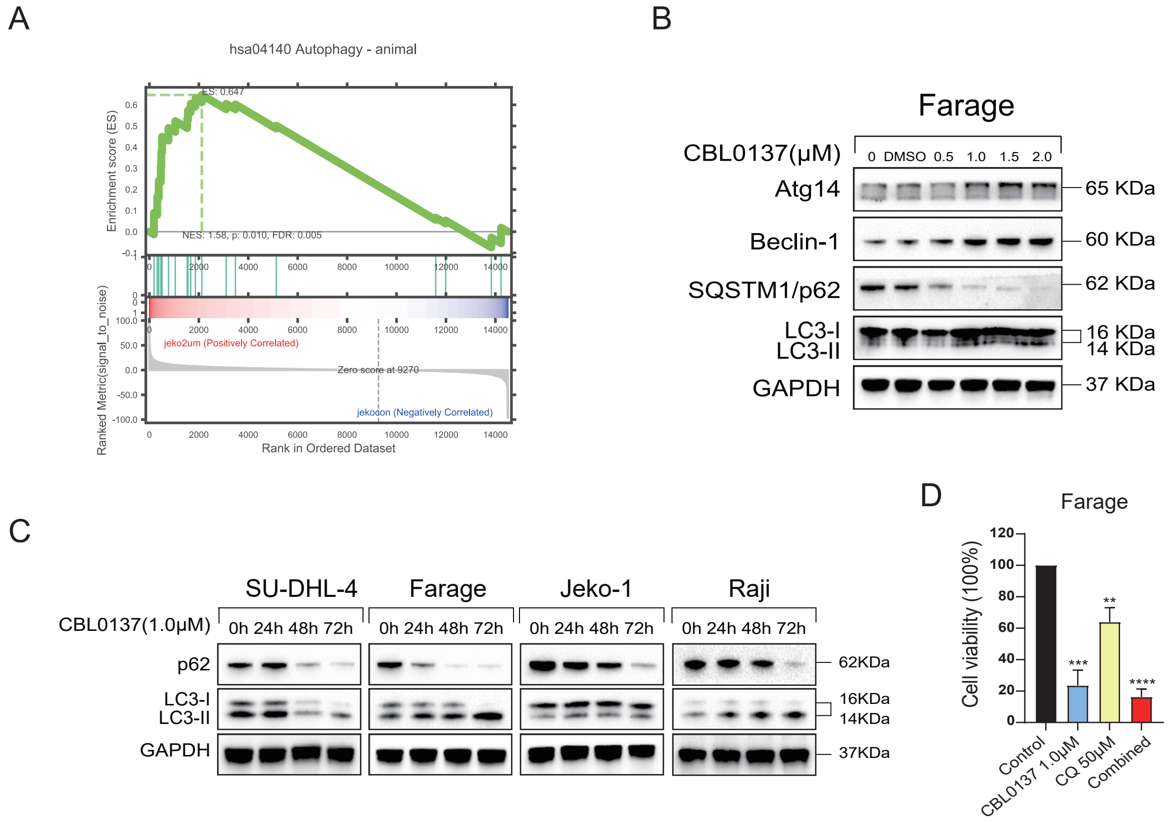

A
hsa04140 Autophagy - animal
ES: 0.647
NES: 1.58, p: 0.010, FDR: 0.005
Enrichment score (ES)
jeko2um (Positively Correlated)
Zero score at 9270
jekocon (Negatively Correlated)
Ranked Metric(signal_to_noise)
Rank in Ordered Dataset
B
Farage
CBL0137(μM)
0 DMSO 0.5 1.0 1.5 2.0
Atg14
65 KDa
Beclin-1
60 KDa
SQSTM1/p62
62 KDa
LC3-I
LC3-II
16 KDa
14 KDa
GAPDH
37 KDa
C
SU-DHL-4
Farage
Jeko-1
Raji
CBL0137(1.0μM)
0h 24h 48h 72h
p62
62KDa
LC3-I
LC3-II
16KDa
14KDa
GAPDH
37KDa
D
Farage
Cell viability (100%)
Control
CBL0137 1.0μM
CQ 50μM
Combined

Fig. S4

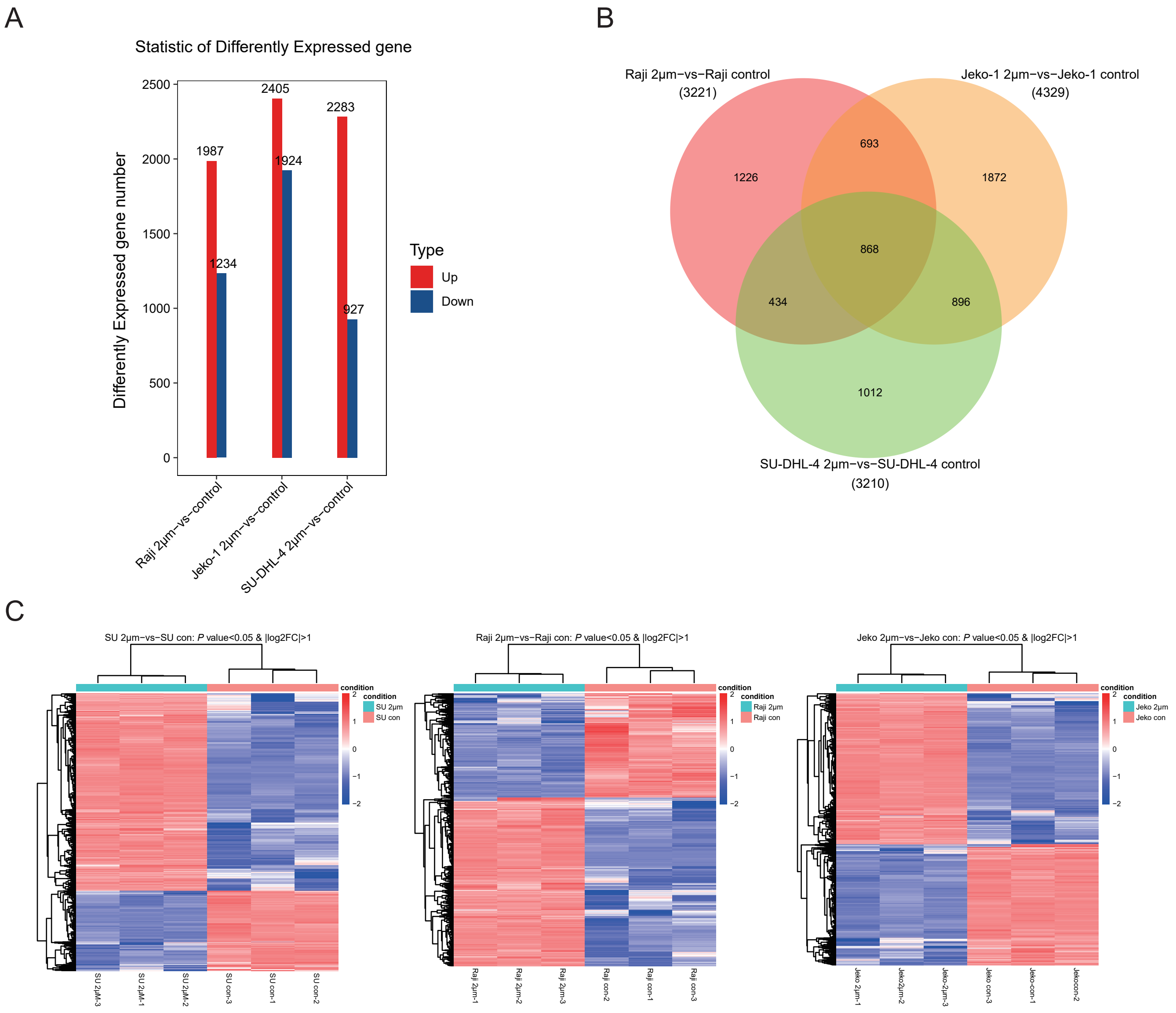
A
Statistic of Differently Expressed gene
Differently Expressed gene number
2500
2000
1500
1000
500
0
1987
1234
2405
1924
2283
927
Raji 2μm−vs−control
Jeko-1 2μm−vs−control
SU-DHL-4 2μm−vs−control
Type
Up
Down
B
Raji 2μm−vs−Raji control
(3221)
Jeko-1 2μm−vs−Jeko-1 control
(4329)
SU-DHL-4 2μm−vs−SU-DHL-4 control
(3210)
1226
693
1872
868
434
896
1012
C
SU 2μm−vs−SU con: P value<0.05 & |log2FC|>1
condition
SU 2μm
SU con
SU 2μM-3
SU 2μM-1
SU 2μM-2
SU con-3
SU con-1
SU con-2
Raji 2μm−vs−Raji con: P value<0.05 & |log2FC|>1
condition
Raji 2μm
Raji con
Raji 2μm-1
Raji 2μm-2
Raji 2μm-3
Raji con-2
Raji con-1
Raji con-3
Jeko 2μm−vs−Jeko con: P value<0.05 & |log2FC|>1
condition
Jeko 2μm
Jeko con
Jeko 2μm-1
Jeko2μm-2
Jeko-2μm-3
Jeko con-3
Jeko-con-1
Jekocon-2
2
1
0
−1
−2

Fig. S5

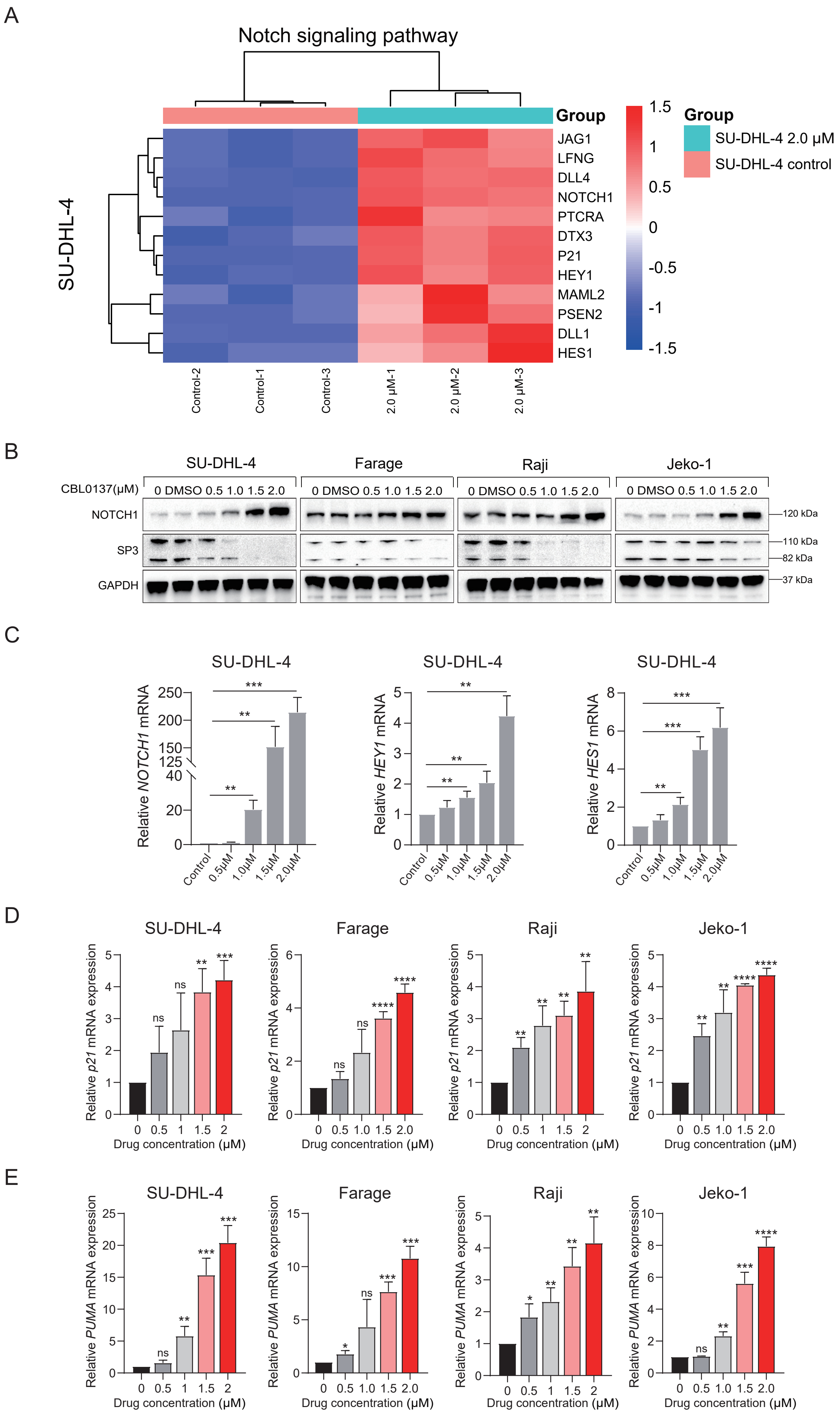
A
Notch signaling pathway
SU-DHL-4
Group
JAG1
LFNG
DLL4
NOTCH1
PTCRA
DTX3
P21
HEY1
MAML2
PSEN2
DLL1
HES1
Control-2
Control-1
Control-3
2.0 μM-1
2.0 μM-2
2.0 μM-3
1.5
1
0.5
0
-0.5
-1
-1.5
Group
SU-DHL-4 2.0 μM
SU-DHL-4 control
B
SU-DHL-4
Farage
Raji
Jeko-1
CBL0137(μM)
0 DMSO 0.5 1.0 1.5 2.0
NOTCH1
SP3
GAPDH
120 kDa
110 kDa
82 kDa
37 kDa
C
SU-DHL-4
Relative NOTCH1 mRNA
Relative HEY1 mRNA
Relative HES1 mRNA
Control
0.5μM
1.0μM
1.5μM
2.0μM
D
SU-DHL-4
Farage
Raji
Jeko-1
Relative p21 mRNA expression
Drug concentration (μM)
E
SU-DHL-4
Farage
Raji
Jeko-1
Relative PUMA mRNA expression
Drug concentration (μM)

Fig. S6

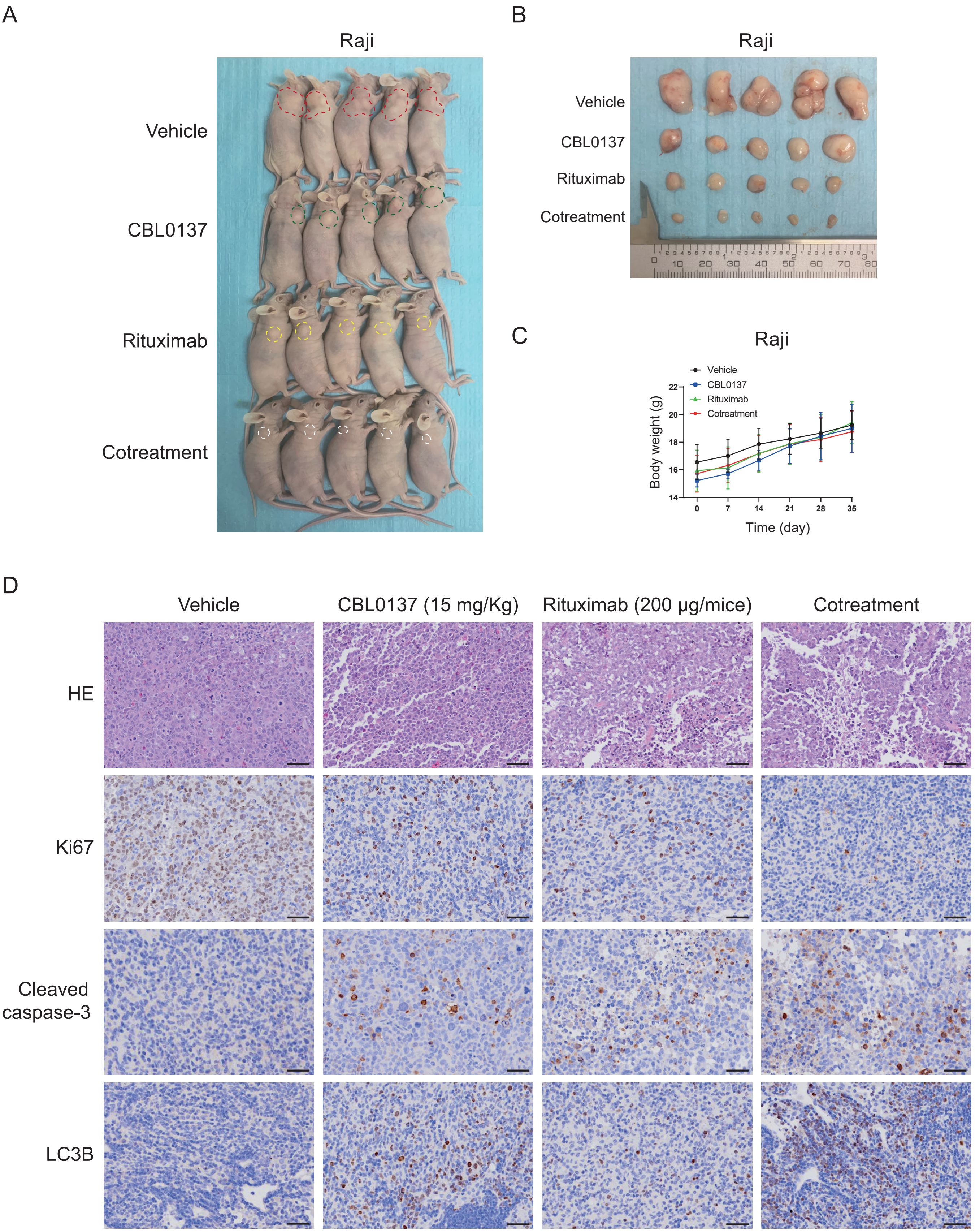
A
Raji
Vehicle
CBL0137
Rituximab
Cotreatment
B
Raji
Vehicle
CBL0137
Rituximab
Cotreatment
C
Raji
Vehicle
CBL0137
Rituximab
Cotreatment
Body weight (g)
14
16
18
20
22
0
7
14
21
28
35
Time (day)
D
Vehicle
CBL0137 (15 mg/Kg)
Rituximab (200 µg/mice)
Cotreatment
HE
Ki67
Cleaved caspase-3
LC3B

Supplementary figure legends

Fig. S1. CBL0137 exerts antitumor activity by inhibiting FACT function and regulating p53 and NF-κB activity. (A) Levels of SSRP1 and SUPT16H mRNA expression in DLBCL and adjacent normal tissue (NT) samples (number in parentheses = number of samples of per type, from TCGA database). (B) Pearson correlation analysis of SSRP1 and SUPT16H in DLBCL. (C) Western blotting experiments showing the protein levels of SSRP1 and SUPT16H in peripheral blood mononuclear cells (PBMC) of a healthy donor and B-NHL cell lines. (D, E) The 24 h-IC50 of CBL0137 in SU-DHL-4, Farage, Raji, and Jeko-1 cells. (F) Changes in OD values of Farage cells were treated with various concentrations of CBL0137 at different time points. (G) Colony assays were conducted in Farage cells. (H) Western blotting of soluble and chromatin containing-containing pellet fractions from B-NHL cells treated with or without CBL0137 for 24 h. (I) Western blotting was used to evaluate the relative expression levels of p53, p-p53, and NF-κB after 24 h treatment with CBL0137.

Fig. S2. CBL0137 induces S phase cell cycle arrest and apoptosis in B-NHL cells. (A) Representative GSEA plots illustrating cell cycle gene sets from Jeko-1 cell line. (B) Four B-NHL cells (SU-DHL-4, Farage, Raji, and Jeko-1) were treated with 0.5 μM, 1.0 μM, 1.5 μM, 2.0 μM CBL0137 or DMSO respectively for 24 h, and the cell cycle distributions were observed. The data shown are representative results from one of three independent experiments. (C) Western blotting was used to detect the changes of apoptosis-related proteins in B-NHL cells treated with 1.0 μM CBL0137 for 24 h, 48 h, and 72 h.

Fig. S3. CBL0137 induces autophagy in human B-NHL cells. (A) Representative GSEA plots illustrating autophagy gene sets from Jeko-1 cell line. (B) Expression of autophagy-related proteins was detected by western blotting in Farage cells treated with different concentrations of CBL0137 or DMSO. (C) The expression levels of LC3I/II and p62 in B-NHL cells treated with 1.0 μM CBL0137 for 24 h, 48 h, and 72 h were detected by western blotting. (D) Farage cells were treated with 1.0 μM CBL0137 either alone or in combination with 50 μM CQ for 24 h, cell viability was detected by CCK-8.

Fig. S4. Differentially expressed genes (DEGs) were analyzed in SU-DHL-4, Raji, and Jeko-1 cell. (A) All up-regulated and down-regulated DEGs were shown with 2.0 μM CBL0137 in SU-DHL-4, Raji, and Jeko-1 compared with control cells. (B) Venn diagram showing overlap DEGs in SU-DHL-4, Raji, and Jeko-1 cells. (C) Heatmap of hierarchical clustering analysis of treated with 2.0 μM of CBL0137 transcriptome from SU-DHL-4, Raji, and Jeko-1 cells.

Fig. S5. CBL0137 targets NOTCH signaling in B-NHL cells. (A) Differentially expression of genes associated with NOTCH signaling pathway in SU-DHL-4 cells treated with or without 2.0 μM CBL0137 for 24 h. The color in the heatmap represents the normalized expression value. (B) NOTCH1 and its negative regulator SP3 protein levels were determined in four B-NHL cells treated with different concentrations of CBL0137 by western blotting. (C~E) B-NHL cells were treated with 0.5 ~ 2.0 μM CBL0137 or DMSO as the control for 24 h. (C) The mRNA expression levels of *NOTCH1* and its transcription-related targets *HEY1* and *HES1*, (D) *p21*, and (E) *PUMA* were determined by qRT-PCR. All values are expressed as mean ± SD (**P* < 0.05, ***P* < 0.01, ****P* < 0.001, *****P* < 0.0001, Student's

t-test). All experiments were repeated three times.

Fig. S6. CBL0137 showed enhanced effects with rituximab in inhibiting the growth of BL xenograft tumors *in vivo*. (A, B) CBL0137 plus rituximab showed more significant antitumor effects than any single treatment alone. (C) The body weight of female mice in four groups was recorded (n=5). (D) H&E and immunohistochemical staining of tumor specimens. Significantly up-regulated cleaved caspase-3 and LC3B expression and significantly decreased Ki67 expression were observed in the co-treatment group compared to the other groups. Scale bars = 50 μm. $^{**}P < 0.01$, $^{***}P < 0.001$, $^{****}P < 0.0001$.

Table S1. Source and identifier of reagents used in this study.

Reagents	Source	Identifier
CBL0137	Selleck Chemicals	Cat# S8483
N-acetylcysteine (NAC)	Selleck Chemicals	Cat# S1623
Chloroquine (CQ)	Selleck Chemicals	Cat# S6999
3-Methyladenine (3-MA)	Selleck Chemicals	Cat# S2726
Z-VAD-FMK	Selleck Chemicals	Cat# S7023
U0126	Selleck Chemicals	Cat# S1102
LY294002	Selleck Chemicals	Cat# S1105
Rituximab	Roche Holding AG	Cat# SH0176

Table S2. Source and identifier of antibodies used in this study.

Antibodies	Source	Identifier
Rabbit monoclonal anti-PARP (46D11)	Cell Signaling Technology	Cat# 9532
Rabbit monoclonal anti-Cleaved PARP (Asp214) (D64E10)	Cell Signaling Technology	Cat# 5625
Rabbit monoclonal anti-Cleaved caspase-9 (Asp330) (D2D4)	Cell Signaling Technology	Cat# 7237
Rabbit monoclonal anti-Cleaved caspase-3 (Asp175)	Cell Signaling Technology	Cat# 9661
Rabbit monoclonal anti-Cleaved caspase-7 (Asp198) (D6H1)	Cell Signaling Technology	Cat# 8438
Rabbit monoclonal anti-Cleaved caspase-8 (Asp374) (18C8)	Cell Signaling Technology	Cat# 9496
Mouse monoclonal anti-Phospho-p53 (Ser15) (16G8)	Cell Signaling Technology	Cat# 9286
Mouse monoclonal anti-P53 (1C12)	Cell Signaling Technology	Cat# 2524
Rabbit monoclonal anti-Phospho-NF-κB p65 (Ser536) (93H1)	Cell Signaling Technology	Cat# 3033
Rabbit monoclonal anti-Bcl-xL (54H6)	Cell Signaling Technology	Cat# 2764
Rabbit monoclonal anti-Puma (D30C10)	Cell Signaling Technology	Cat# 12450
Rabbit monoclonal anti-Bax (D2E11)	Cell Signaling Technology	Cat# 5023
Rabbit monoclonal anti-Beclin-1 (D40C5)	Cell Signaling Technology	Cat# 3495
Rabbit monoclonal anti-LC3A/B (D3U4C)	Cell Signaling Technology	Cat# 12741

Rabbit monoclonal anti-Phospho-PI3 Kinase p85 (Tyr458)/p55 (Tyr199)	Cell Signaling Technology	Cat# 4228
Rabbit monoclonal anti-PI3 Kinase p110α (C73F8)	Cell Signaling Technology	Cat# 4249
Rabbit monoclonal anti-Phospho-Akt (Ser473) (D9E)	Cell Signaling Technology	Cat# 4060
Rabbit monoclonal anti-Akt (pan) (C67E7)	Cell Signaling Technology	Cat# 4691
Rabbit monoclonal anti-Phospho-mTOR (Ser2448) (D9C2)	Cell Signaling Technology	Cat# 5536
Rabbit monoclonal anti-mTOR (7C10)	Cell Signaling Technology	Cat# 2983
Rabbit monoclonal anti-Phospho-p44/42 MAPK (Erk1/2) (Thr202/Tyr204) (D13.14.4E)	Cell Signaling Technology	Cat# 4370
Rabbit monoclonal anti-p44/42 MAPK (Erk1/2) (137F5)	Cell Signaling Technology	Cat# 4695
Rabbit monoclonal anti-Phospho-p38 MAPK (Thr180/Tyr182) (D3F9)	Cell Signaling Technology	Cat# 4511
Rabbit monoclonal anti-p38 MAPK (D13E1)	Cell Signaling Technology	Cat# 8690
Rabbit monoclonal anti-Notch1 (D6F11)	Cell Signaling Technology	Cat# 4380
Rabbit monoclonal anti-GAPDH (D4C6R)	Cell Signaling Technology	Cat# 97166
Rabbit monoclonal anti-SSRP1 [EPR7894]	Abcam	Cat# ab129109
Rabbit monoclonal anti-p21 [EPR362]	Abcam	Cat# ab109520
Rabbit monoclonal anti-Cyclin D1 [EPR2241]-C-terminal	Abcam	Cat# ab134175
Rabbit monoclonal anti-Cyclin B1 [Y106]	Abcam	Cat# ab32053

Rabbit monoclonal anti-Cyclin E1 [EPR194]	Abcam	Cat# ab133266
Rabbit monoclonal anti-Cyclin A2 [Y193]	Abcam	Cat# ab32386
Rabbit monoclonal anti-CDK2 [E304]	Abcam	Cat# ab32147
Rabbit monoclonal anti-CDK1 [EPR165]	Abcam	Cat# ab133327
Rabbit monoclonal anti-Cytochrome C [EPR1327]	Abcam	Cat# ab133504
Rabbit monoclonal anti-ICAD [EPR4220]	Abcam	Cat# ab108924
Rabbit monoclonal anti-SQSTM1/p62 [EPR4844]	Abcam	Cat# ab109012
Rabbit monoclonal anti-GSK3 beta [Y174]	Abcam	Cat# ab32391
Rabbit monoclonal anti-GSK3 beta (phospho S9) [EPR2286Y]	Abcam	Cat# ab75814
Rabbit monoclonal anti-SP3 [EPR6806]	Abcam	Cat# ab129099
Rabbit polyclonal anti-SUPT16H	Proteintech	Cat# 20551-1-AP
Mouse monoclonal anti-c-MYC	Proteintech	Cat# 67447-1-Ig
Rabbit polyclonal anti-DR5	Proteintech	Cat# 15497-1-AP
Rabbit polyclonal anti-Ki67	Proteintech	Cat# 27309-1-AP
Rabbit polyclonal anti-DFFB	ABclonal	Cat# A10110
Rabbit polyclonal anti-ATG14	ABclonal	Cat# A7526

Table S3. The primers sequences for qRT-PCR in this study.

Primers	Forward	Reverse
NOTCH1	5’-TCCACCAGTTTGAATGGTCAAT-3’	5’- CGCAGAGGGTTGTATTGGTTC-3’
SP3	5’- GTTGAGGCATTTGGGTGGTA-3’	5’- GGGAGACATGGTTTTTGGAA-3’
HES1	5’- AACACTGATTTTGGATGCTCTG-3’	5’- CACTGTCATTTCCAGAATGTCC-3
HEY1	5’- CTTCCACTTACTGTCTCCCAAT-3’	5’- TTAAAAAGCTCCGATCTCCGTC-3’
P21	5’- CGATGGAACTTCGACTTTGTCA-3’	5’- GCACAAGGGTACAAGACAGTG-3’
PUMA	5’- CCTCAACGCACAGTACGA-3’	5’- CCCATGATGAGATTGTACAGGA-3’
GAPDH	5’-GGATTTGGTCGTATTGGGCG-3’	5’- ATCGCCCCACTTGATTTTGG-3’
